# BEAF Regulates Cell-Cycle Genes through the Controlled Deposition of H3K9 Methylation Marks into Its Conserved *Dual-Core* Binding Sites

**DOI:** 10.1371/journal.pbio.0060327

**Published:** 2008-12-23

**Authors:** Eldon Emberly, Roxane Blattes, Bernd Schuettengruber, Magali Hennion, Nan Jiang, Craig M Hart, Emmanuel Käs, Olivier Cuvier

**Affiliations:** 1 Physics Department, Simon Fraser University, Burnaby, British Columbia, Canada; 2 CNRS, Laboratoire de Biologie Moléculaire Eucaryote, Université de Toulouse, UPS, France; 3 Department of Biological Sciences, Louisiana State University, Baton Rouge, Lousiana, United States of America; 4 Institut de Genetique Humaine, Department of Genome Dynamics, CNRS, Montpelier, France; National Cancer Institute, United States of America

## Abstract

Chromatin insulators/boundary elements share the ability to insulate a transgene from its chromosomal context by blocking promiscuous enhancer–promoter interactions and heterochromatin spreading. Several insulating factors target different DNA consensus sequences, defining distinct subfamilies of insulators. Whether each of these families and factors might possess unique cellular functions is of particular interest. Here, we combined chromatin immunoprecipitations and computational approaches to break down the binding signature of the *Drosophila* boundary element–associated factor (BEAF) subfamily. We identify a dual-core BEAF binding signature at 1,720 sites genome-wide, defined by five to six BEAF binding motifs bracketing 200 bp AT-rich nuclease-resistant spacers. Dual-cores are tightly linked to hundreds of genes highly enriched in cell-cycle and chromosome organization/segregation annotations. siRNA depletion of BEAF from cells leads to cell-cycle and chromosome segregation defects. Quantitative RT-PCR analyses in BEAF-depleted cells show that BEAF controls the expression of dual core–associated genes, including key cell-cycle and chromosome segregation regulators. *beaf* mutants that impair its insulating function by preventing proper interactions of BEAF complexes with the dual-cores produce similar effects in embryos. Chromatin immunoprecipitations show that BEAF regulates transcriptional activity by restricting the deposition of methylated histone H3K9 marks in dual-cores. Our results reveal a novel role for BEAF chromatin dual-cores in regulating a distinct set of genes involved in chromosome organization/segregation and the cell cycle.

## Author Summary

The genome of eukaryotes is packaged in chromatin, which consists of DNA, histones, and accessory proteins. This leads to a general repression of genes, particularly for those exposed to mostly condensed, heterochromatin regions. DNA sequences called chromatin insulators/boundary elements are able to insulate a gene from its chromosomal context by blocking promiscuous heterochromatin spreading. No common feature has been identified among the insulators/boundary elements known so far. Rather, distinct subfamilies of insulators harbor different DNA consensus sequences targeted by different DNA-binding factors, which confer their insulating activity. Determining whether distinct subfamilies possess distinct cellular functions is important for understanding genome regulation. Here, using *Drosophila*, we have combined computational and experimental approaches to address the function of the boundary element-associated factor (BEAF) subfamily of insulators. We identify hundreds of BEAF dual-cores that are defined by a particular arrangement of DNA sequence motifs bracketing nucleosome binding sequences, and that mark the genomic BEAF binding sites. BEAF dual-cores are close to hundreds of genes that regulate chromosome organization/segregation and the cell cycle. Since BEAF acts by restricting the deposition of repressing epigenetic histone marks, which affects the accessibility of chromatin, its depletion affects the expression of cell-cycle genes. Our data reveal a new role for BEAF in regulating the cell cycle through its binding to highly conserved chromatin dual-cores.

## Abstract 3

Chromatin Dual-Cores define new potent nucleosome-associated*cis*-regulatory elements that regulate the accessibility of promoters of genes controlling chromosome organization/segregation and the cell cycle.

## Introduction

Chromatin insulators/boundary elements (BEs) [1,2] are defined as sequences able to insulate a transgene from its chromosomal context and to block promiscuous enhancer–promoter interactions or heterochromatin spreading [1,3–5]. These elements are thought to subdivide the genome into functional chromosome domains, through their ability to cluster DNA loops [1,2] and to control the deposition of histone epigenetic marks [6–8] to regulate chromatin accessibility for gene expression [9–13].

No common signature and/or mechanism of action has been identified among characterized insulators/boundary elements [2]. Rather, several factors confer insulating activity by targeting different DNA consensus sequences in the known insulators. In *Drosophila*, insulating factors include dCTCF [14,15], Zw5 [16], boundary element–associated factor (BEAF) [17], and the well-characterized suppressor of Hairy-wing (Su(Hw)) [1,18,19], which targets hundreds of distinct, largely uncharacterized genomic sites [20–22]. Whether each of these factors and subfamily of insulators might possess distinct cellular functions is of particular interest.

BEAF blocks both enhancer–promoter communication [17,23–25] and repression by heterochromatin, as shown using reporter transgenes [5,25]. This insulating activity of BEAF was also evidenced by a genetic screen in *yeast* [4], confirming that, unlike de-silencing activity, BEAF binding sites must bracket a transgene for insulation. The hundreds of BEAF binding sites have not been characterized in situ, however, and the cellular function of BEAF remains to be elucidated in vivo.

Here we have combined computational and experimental approaches to address the function of BEAF binding sites in vivo. We have identified ≈1,720 BEAF *dual-core* elements genome-wide that share an unusual organization conserved over 600 bp. The dual-core signature consists of five to six BEAF binding motifs bracketing 200 bp AT-rich nuclease-resistant spacers. BEAF dual-cores juxtapose to hundreds of genes highly enriched in gene annotations regulating chromosome organization/segregation and the cell cycle. Accordingly, BEAF depletion leads to cell-cycle and chromosome segregation defects. Quantitative RT-PCR analyses further show that dual-cores regulate the expression of key cell-cycle genes including *cdk7* and *mei-S332*. These results are also reproduced in embryos expressing truncated *beaf* mutants, which abolish the proper targeting of BEAF to dual-cores and its insulating activity. Chromatin immunoprecipitation analyses show that BEAF acts by restricting the deposition of methylated H3K9 marks in dual-cores. Our data reveal a new role for BEAF in regulating chromosome organization/segregation and the cell cycle through its binding to highly conserved chromatin dual-cores.

## Results

### Breaking Down the Binding Code of BEAF to Dual-Cores

**Figure 1:**
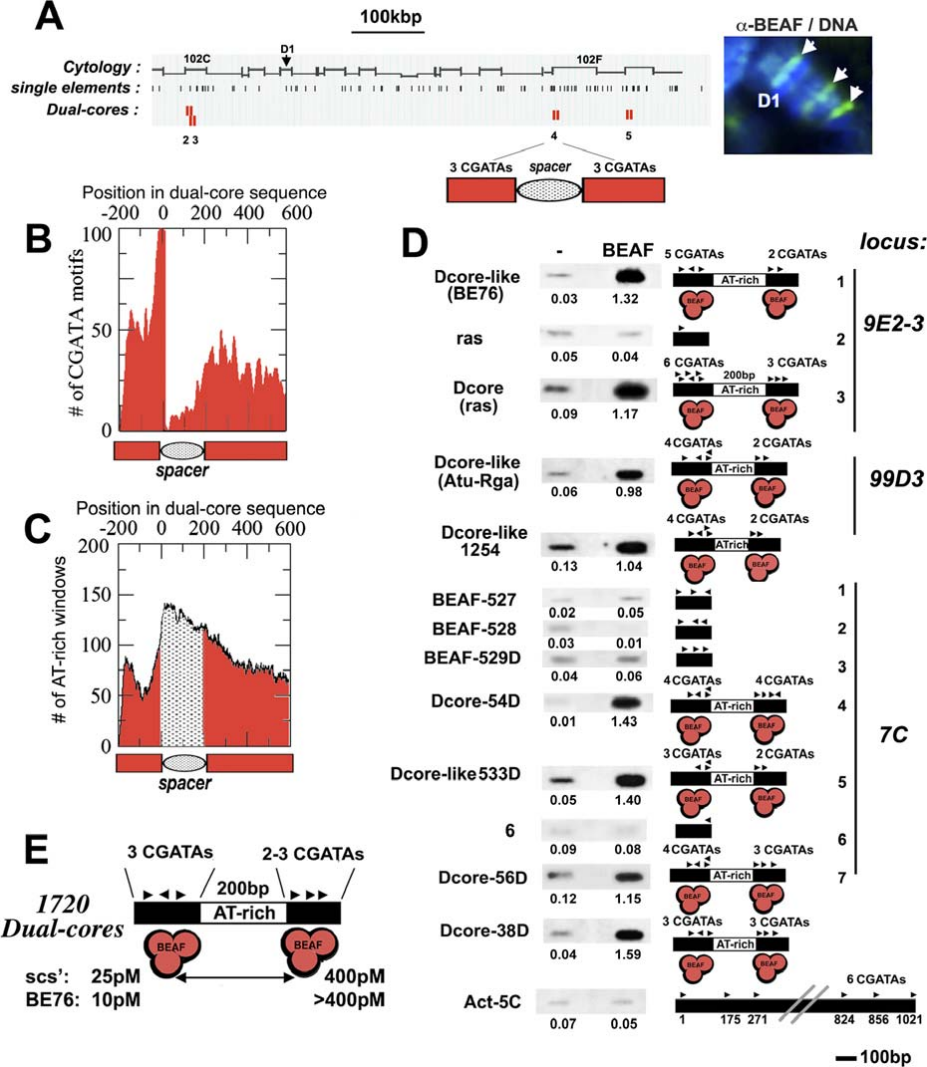
BEAF *Dual-Cores* Define a New Class/Family of Chromatin Elements (A) BEAF single-element and dual-core predictions are shown in parallel with immunostaining with anti-BEAF antibodies for Chromosome 4 (1.2 Mbp; (A); D1 = recognizable band of Chromosome 4). Each single element contains ≥3 BEAF CGATA consensus motifs within 200 bp, and each dual-core corresponds to two juxtaposed single BEAF elements (see text). (B,C) Statistical analysis of dual-cores. (B) Shows the distribution of 12,058 CGATA motifs of dual-cores into two clusters (3CGATAs × 2) separated by a CGATA-free spacer. (C) Shows the localization of AT-rich 200-bp windows (>70% A+T) in the spacer. Position 0 is the location of the right-most CGATA motif in the first cluster of dual-cores. This analysis includes dual core–*like* elements, which contain two (instead of three) CGATAs within 100 bp in the second cluster. (D) ChIP analysis with anti-BEAF or control IgG antibodies on DNA sequences corresponding to the indicated dual-cores or control regions. CGATAs are represented by arrowheads. Numbers below each blot represent the percentage of immunoprecipitated DNA over input genomic DNA as standard. (E) The BEAF dual-core signature.

The DNA-binding activity of BEAF has been well-characterized in vitro [17,20,23,24]. Each subunit of the BEAF complex targets one CGATA motif. Point mutations within this consensus abolish both its binding and insulating activities. Clusters of three to four CGATA motifs can create high-affinity (K_d_ ∼ 10–25 pM) BEAF in vitro binding sites, which we call single elements. A computational scan of the *Drosophila* genome revealed thousands of single elements, yet immunostaining analysis demonstrated that they were not good predictors for BEAF binding in vivo. For example, Chromosome 4 was found to contain hundreds of single elements, yet immunodetection analysis showed only three major BEAF signals on this chromosome (Figure 1A). Interestingly, statistical analysis showed that single elements were often organized in a pair-wise configuration. Genome-wide, 988 single elements form 494 so-called “dual-cores,” which harbor two separate clusters of three CGATAs, a statistically significant result (*p*-value ∼ 1e-9). Moreover, 1,226 additional “dual-core–like” elements have a second cluster of two (instead of three) CGATAs. These elements include all characterized BEAF insulators whose activity involves a second, lower-affinity CGATA cluster (K_d_ ∼ 400–600 pM) where BEAF binding is abolished when the first high-affinity cluster is mutated [20,23].

Detailed analysis by alignment of all 1,720 dual-core and dual-core–like elements showed a highly organized distribution of their 12,058 CGATAs, which preferentially segregate into two clusters separated by spacers of approximately 200 bp (Figure 1B). For *scs'* and other characterized BEAF insulators, these spacers were found to be relatively AT-rich [20,24,26]. Scanning the 1,720 dual-cores for A+T content showed that they all harbor significant AT-rich (>70%) sequences in their spacers (Figure 1C, Figure S1). The remarkably conserved organization of dual-cores indicates that they likely correspond to a highly specific BEAF-binding signature.

**Figure 2:**
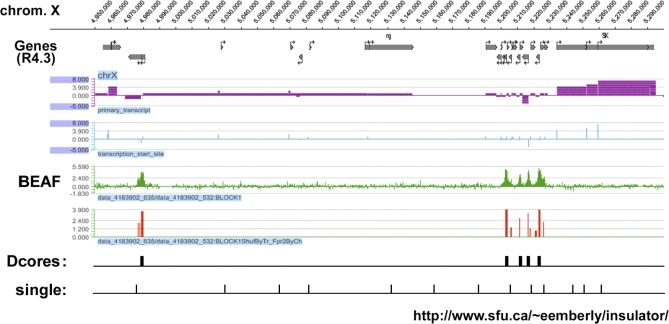
ChIP-on Chip Analysis Shows That BEAF Peaks Corresponding to *Dual-Cores* The panel shows an alignment of ChIP-on-chip analysis using anti-BEAF antibodies (see the graph in green with red bars marking the position of significant peaks), with our dual-core database (Dcores, black rectangles) (http://www.sfu.ca/~eemberly/insulator/), over a region of the X chromosome (nucleotide positions 4,950,000 to 5,300,000). Single elements not forming dual-cores are also shown (single:, black bars). Note that all five peaks fit into dual-cores. The second peak from the left is X-Dcore38_D, which juxtaposes the *cdk7* gene. Transcription start sites (blue bars) and primary transcripts (purple lines) are shown on top.

We tested this possibility by assaying BEAF binding to dual-cores by chromatin immunoprecipitation (ChIP) and ChIP-on-chip (Figures 1D and 2). Based on the signals obtained with anti-BEAF antibodies, dual-cores are expected to be precipitated much more efficiently than single elements (Figure 1A). Indeed, ChIP analysis confirmed that single elements were not bound by BEAF (Figure 1D). In contrast, dual-cores from the 7C locus of the X chromosome were efficiently bound by BEAF (Figure 1D, probes 4 and 5), while nearby control sequences or single elements were not (probes 1, 2, 3, and 6). Altogether, 25 out of 25 dual-cores and dual-core–like elements assayed by ChIP were found to be efficiently bound by BEAF in vivo (Figures 1D and 2; unpublished data). The *actin* promoter region, which contains six unclustered CGATA motifs, was not bound by BEAF (Figure 1D; last row), indicating that the distribution of CGATAs in dual-cores, rather than the number of CGATAs *per se*, is important for BEAF binding. Furthermore, ChIP-on-chip analysis over 350 Kbp of the X chromosome strengthens our conclusions, as all major peaks corresponding to regions where BEAF binds in vivo fit into a dual-core or a dual-core–like element (hereafter called “dual-cores”, see black rectangles in Figure 2; see our database at http://www.sfu.ca/~eemberly/insulator/ for a complete listing). We note that computer analysis occasionally retrieved minor peaks present in the shoulder of the major BEAF peaks (enrichment <2; red bars in Figure 2) that may be attributed to the cooperative binding of BEAF to additional CGATA motifs present in single elements juxtaposed to dual-cores (Figure 2, see black bars for “single”). However, no peaks were present in regions corresponding to dispersed single elements (Figure 2; see our database), confirming that they are not sufficient for BEAF binding. These results establish that BEAF elements organized into dual-cores indeed define a characteristic in vivo BEAF-binding signature (Figure 1E).

### BEAF Dual-Cores Are Tightly Linked to a Discrete Set of Gene Ontologies

**Figure 3:**
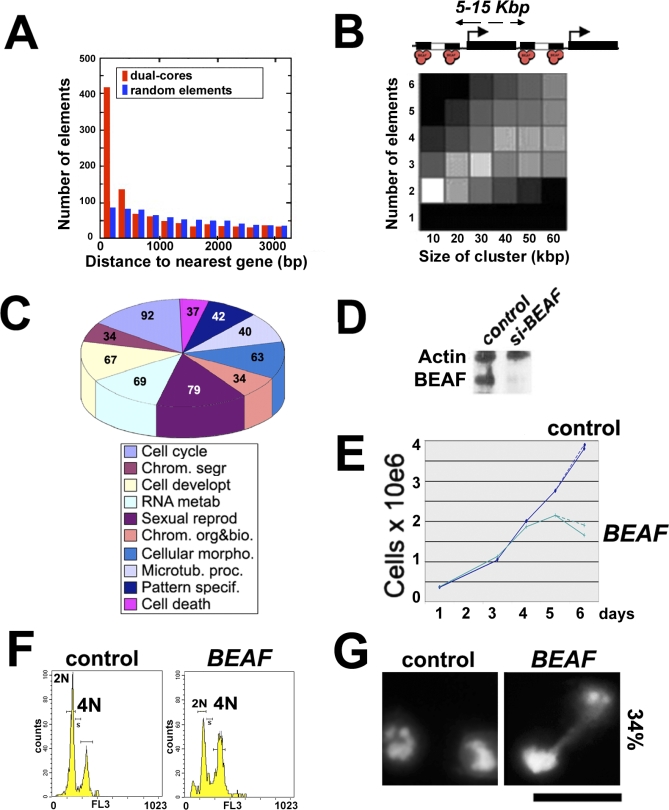
Genomic Features Associated with BEAF Dual-Cores (A) Distribution of the distances between TSSs and dual-cores (red) compared to a theoretical distribution for randomly placed elements (blue) (see Materials and Methods). *y*-Axis, number of dual-cores; *x*-axis, distance to the nearest gene (in bp). (B) Statistical analyses of the distribution of dual-cores. Dual-cores are preferentially organized in pairs separated by 5–15 Kbp as depicted on top (white square: *p*-value = 1.01e-33) most likely due to the organization of genes in clusters (see text). *y*-Axis, number of dual-cores; *x*-axis, distance between two dual-cores. Lighter (/darker) boxes represent the most (/less) significant *p*-values (see Materials and Methods). (C) Pie chart showing the most statistically significant (*p*-value < 10e-6) GOs for dual-core-associated genes (<500 bp of TSS) (see Materials and Methods). (D) Western blotting of control or *beaf* siRNA-treated SL2 cells using anti-actin (dual-core–free gene) (see Figure 1D) and anti-BEAF antibodies. (E–G) Cell growth (E), FACS (F), and microscopy (G) analyses performed in parallel from the same BEAF-depleted or control cell samples shown in (D). In (E), the two curves for BEAF-depleted cells show the standard variation from two independent experiments. In (F), the *y*-axis indicates the number of cells (counts) and the *x*-axis the FL3 channel used to measure the staining of nuclei with propidium iodide. 4N indicates the number of G2/M cells with 4N DNA content. In (G), 34% indicates the increase in the percentage of BEAF-depleted cells with apparent chromosome segregation defects compared to control cells. DNA was stained with Hoechst. Bar, 10μm.

Analysis of the positioning of dual-cores relative to genes showed that they are preferentially associated with gene-dense regions. 545 dual-cores reside within 500 bp of promoter/transcriptional start sites (TSSs) (*p*-value = 6.7e-119) (Figure 3A), and more than 850 are within 2,000 bp. As dual-cores are preferentially distributed in pairs separated by approximately 5–15 kbp (*p*-value = 1.01e-33; Figure 3B), the remaining elements might be found at the 3′ borders of genes. However, we could not find any specific enrichment for dual-cores in the 3′ UTR of genes (unpublished data), indicating that the clustering of dual-cores can be attributed to the clustering of genes/TSS rather than the bracketing of genes by dual-cores per se. These features (see our genome-wide database) raise the possibility that dual-cores might exert a function distinct from that of Su(Hw) binding sites, which rarely juxtapose the TSS of genes [21,22,27].

Strikingly, genes containing a dual-core near their promoter were statistically enriched in gene-class ontology (GO) groups that include the cell cycle, chromosome organization/segregation, apoptosis, and sexual reproduction (*p*-value < 1e-6; Figure 3C). These essential cellular processes require constitutive regulation, whereas genes associated with non-constitutive processes such as sensing and behavior were not enriched for BEAF dual-cores (Table S1). Inspection of Table S1 also shows that other cell functions enriched in BEAF dual-cores include GOs that can be linked to phenotypes observed in *beaf* mutants [25,28,29], such as chromosome architecture, germ-cell and imaginal-disc development, and eye morphogenesis defects. We asked whether BEAF might be involved in regulating the cell-cycle and/or chromosome organization by siRNA-mediated depletion of BEAF from cells. Reduction of BEAF levels to background occurred from day 3–4 (Figure 3D), when defects in cell growth are first observed (Figure 3E). In addition, FACS and microscope analyses showed that BEAF depletion led to a significant and reproducible increase (>3×) in the proportion of cells with 4N DNA content and with phenotypes typical of chromosome segregation defects (Figure 3F and 3G). These observations support our conclusion that the selective association of the corresponding GOs with closely linked dual-cores likely reflects a biologically significant localization.

### BEAF Dual-Cores Control the Expression of Key Cell-Cycle Regulators

**Figure 4:**
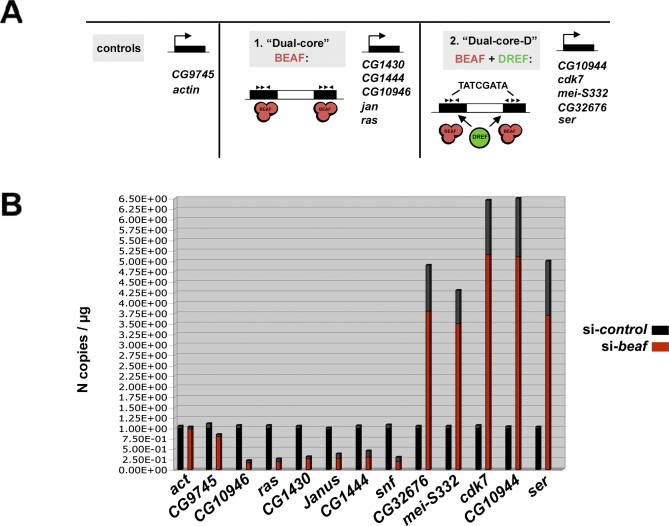
DREF Modulates the Activity of BEAF Dual-Cores Associated with Key Cell-Cycle Genes (A) Schematic representation of the two distinct classes of BEAF dual-cores depending on the presence of an additional consensus (TATCGATA) for the transcription factor DREF (denoted “Dual-core_D”; see our database and text). (B) Quantitative RT-PCR analysis of the expression of a control gene (left; *actin*, *CG9745*), of dual core-associated genes (without a TATCGATA DREF element, middle; *CG1430*, *CG1444*, *CG10946*, *janus* (*jan*), *rasberry* (*ras*)) or of dual core_D–associated genes (with a DREF element, right; *CG10944*, *cdk7*, *mei-S332*, *CG32676*, *serendipity* (*ser*)) in BEAF-depleted (red bars) or control cells (black bars). The *y*-axis shows the number of copies of amplification products per μg of RNA normalized for each gene in control cells (see Materials and Methods), where N =1 corresponds to 8,450 copies of *actin*, 14,930 copies of *CG9745*, 29,300 copies of *CG10946*, 24,800 copies of *ras*, 23,020 copies of *CG1430*, 43,020 copies of *janus*, 29,370 copies of *CG1444*, 80,390 copies of *snf*, 48,393 copies of *CG32676*, 56,300 copies of *mei-S332*, 114,500 copies of *cdk7*, 32,830 copies of *CG10944*, and 49,620 copies of *serendipity*. The copy number was estimated using a standard curve obtained from more than three different quantities of genomic DNA. Experimental error from three independent experiments is denoted by the differentially colored (gray) portion at the top of each bar.

We next asked whether the phenotypes observed upon BEAF-depletion can be attributed to the loss of activity of BEAF dual-cores associated with 160 genes that control cell-cycle chromosome dynamics. These include *mei-S332* and *cdk7*, two major chromosome-segregation and cell-cycle regulators [30–32] whose promoter regions are bound by BEAF in vivo (Dual-cores 38/56, Figure 1D). Remarkably, further DNA-motif searches showed that the dual-cores associated with *cdk7* and *mei-S332*, and more generally with all genes belonging to the cell-cycle and/or chromosome dynamic GOs, also contain the TATCGATA consensus sequence recognized by DREF (*p*-value ∼ 2.4e-6; Figure 4A). DREF activates hundreds of cell-cycle regulatory genes [33] and, importantly, might compete with BEAF for binding to the overlapping consensus [34]. Hence, DREF-regulated dual-cores may define a distinct regulatory subclass (Figure 4A, right).

To test how BEAF might affect the expression of genes associated with dual-cores that do or do not contain a DREF consensus site, we performed quantitative RT-PCR expression analysis from BEAF-depleted or control cells (Figure 4). BEAF depletion did not affect the expression of control genes (see Figure 4A, left), including those located near a single element (Figure 4B; *actin*, *CG9745*) where BEAF does not bind (Figure 1). The expression of all genes associated with a dual core lacking a DREF element was consistently found to be positively regulated by BEAF by approximately 4-fold to 5-fold (*CG1430, CG10946, CG1444, snf, ras*, *janus*; Figure 4B). These data are in complete agreement with previous work showing that BEAF has a positive effect on gene expression by de-repressing a transgene from surrounding chromatin [17,20,23,24]. In stark contrast, the expression of all genes associated with a dual-core harboring a DREF consensus, including *cdk7* and *mei-S332*, specifically increased by approximately 4- to 6-fold upon depletion of BEAF (Figure 4B; *CG32676*, *mei-S332*, *cdk7*, *CG10944*, *ser*). Accordingly, Western blot analysis showed that Cdk7 and Mei-S332 protein levels increased under these conditions (Figure S2). Therefore, two categories of dual-cores may be found. In those lacking a DREF consensus, BEAF positively regulates gene expression; in those that contain a DREF consensus, BEAF may prevent binding of DREF to its overlapping consensus, thereby controlling the activation of the associated cell-cycle and chromosome organization/segregation GOs.

### Mutagenesis of the DREF Site from DREF Binding Dual-Cores Reveals the Positive Effect of BEAF

**Figure 5:**
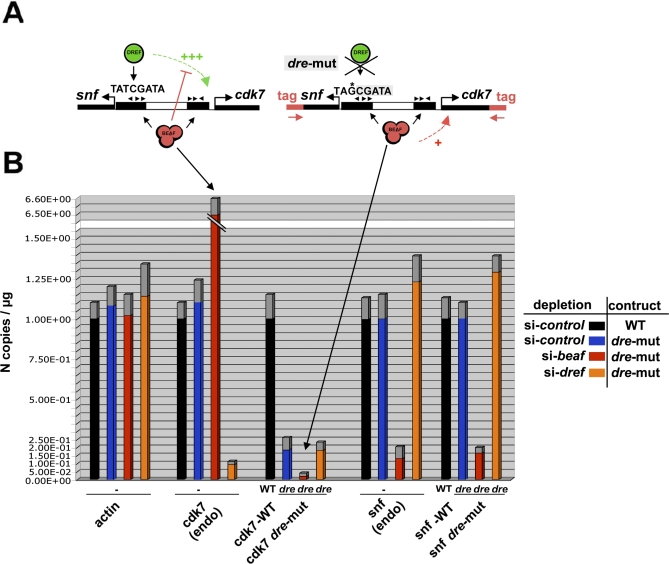
BEAF Dual-Cores Have a Positive Effect on Gene Expression Independent of DREF (A) Schematic representation of the transfected constructs potentially regulated by BEAF–DREF competition for binding to the consensus TATCGATA present in the dual-core_D associated with *cdk7* and *snf* (X_Dcore 38D). Both the wild-type (left) and its DREF-response element (*dre*) mutant form (right) are shown. The transfected Dual-core harbors an additional sequence (tag) for specific amplification by Q-PCR. The +++ sign indicates the activation of *cdk7* by DREF, and the + sign represents the positive effect of BEAF obtained in the absence of the DREF binding site (B). (B) Quantitative RT-PCR analysis in control (black and blue bars), BEAF-depleted (red bars), or DREF-depleted cells (orange bars) of the expression of endogenous genes (*actin*; *cdk7*-*endo*; *snf*-*endo*) and of transfected genes associated with the wild-type (WT) or the mutated version (*dre*; without DREF binding site) of Dcore_38D, as indicated. The *y*-axis shows the number of copies of amplification products per μg of RNA normalized for each gene in control cells, where the copy number N =1 corresponds to 9,680 copies of *actin*, 93,690 copies of *snf*, 138,900 copies of *cdk7*. Note that the expression of the transgenes (WT or *dre*) and of endogenous genes was measured from the same batch of cells. For transfected constructs, gene expression was normalized to the DNA copy-number in the input (for details, see Materials and Methods). Experimental error is denoted by the differentially colored (gray) portion at the top of each bar.

Quantitative RT-PCR analysis showed that DREF depletion resulted in a more than 10-fold down-regulation of *cdk7* (Figure 5), confirming the role of DREF as a transcriptional activator of this gene. To further characterize the respective roles of BEAF and DREF in regulating cell-cycle regulatory genes by binding to dual-cores, we eliminated the DREF consensus from the dual-core associated with *cdk7* (*dre* mutant, Figure 5A) and transfected this construct or its wild-type version into cells depleted of BEAF or of DREF by siRNA (Figure 5B). Because the *dre* mutant does not modify the CGATA BEAF consensus and still harbors the dual-core signature (2× 3CGATAs separated by the spacer; Figure S7B), this construct may be used to reveal the effect of the BEAF dual-core on the expression of *cdk7* independently of DREF. Importantly, mutating the DREF consensus site led to a down-regulation of *cdk7* (Figure 5B, *cdk7-mut*, blue bar), similar to what is found by depleting DREF. Strikingly, BEAF depletion further impaired the expression of *cdk7* by approximately 5-fold (Figure 5B, *cdk7-mut*, red bar) compared to the expression of the identical construct in control cells (Figure 5B, *cdk7-mut*, blue bar). We conclude that, although BEAF regulates DREF-mediated activation, it additionally positively regulates the expression of *cdk7*, as found for other genes associated with a dual-core lacking a DREF consensus. In support of this conclusion, we obtained a similar result for *snf*, which is transcribed in opposite orientation relative to *cdk7* (Figure 5A). *Snf* is under the influence of the same dual-core as *cdk7*, yet its expression is not regulated by DREF (Figure 5B). However, BEAF depletion reproducibly impaired *snf* expression by approximately 6-fold, similar to what we obtained for *cdk7* in the absence of DREF. These results show that BEAF has a positive role on the expression of genes associated with dual-cores, in addition to its role in controlling activation by DREF.

### BEAF Restricts the Deposition of H3K9me3 in Dual-Cores

**Figure 6:**
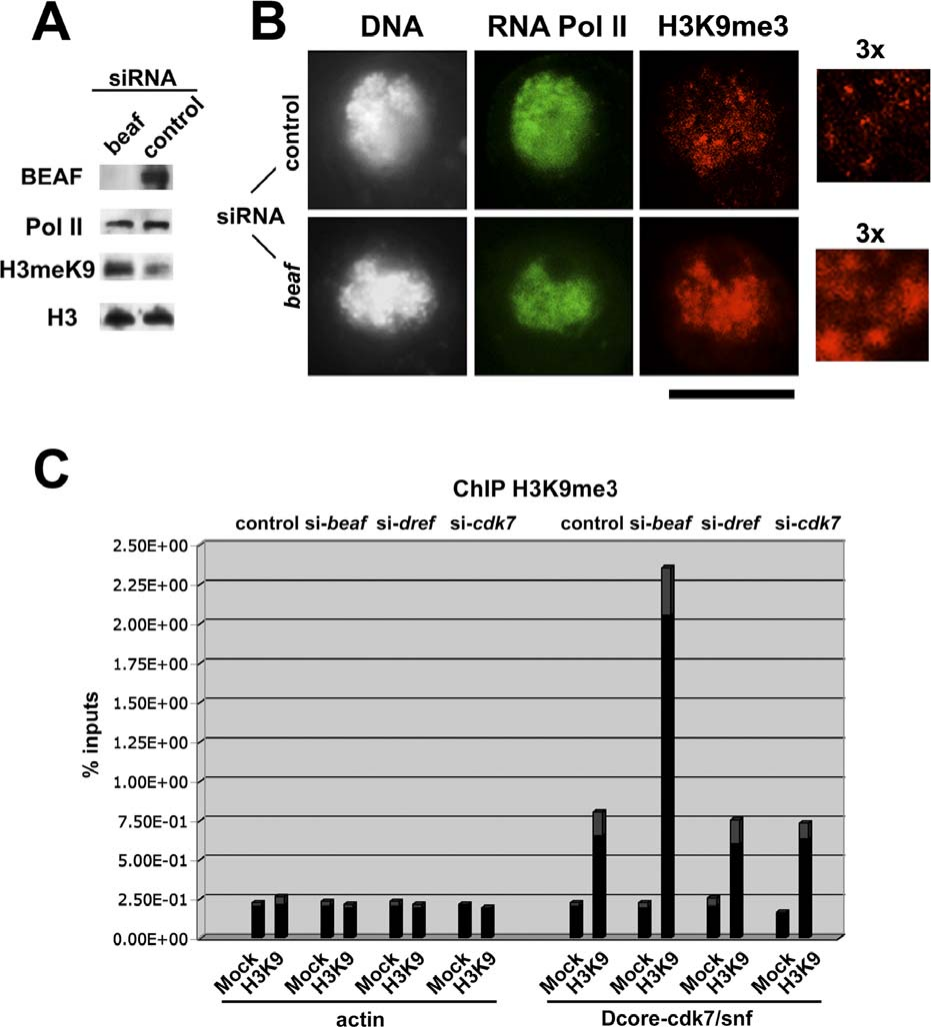
BEAF Restricts the Deposition of H3K9me3 Marks in Dual-Cores (A,B) Western blot (A) and immunostaining (B) analysis with the indicated antibodies of chromatin-associated proteins in BEAF-depleted or control cells. Enlargements of confocal images are also shown (3×). Bar, 10μm. (C) Quantitative PCR analysis following ChIP with mock control or with anti-H3K9me3 (‘H3K9') antibodies in control (left), BEAF-depleted (second from left), DREF-depleted (second from right), or CDK7-depleted (right) cells. The graph shows the analysis of the *actin* control region and of dual-core_38D associated with *cdk7-snf*. Note that similar results were obtained for the dual-cores associated with *mei-S332* (unpublished data). The *y*-axis shows the percentage of material precipitated from inputs (see Materials and Methods for details). Experimental error is denoted by the differentially colored (gray) portion at the top of each bar.

BEAF insulating activity can protect a transgene from repression by chromatin [5,25]. The expression of genes positively regulated by dual-cores might implicate mechanisms similar to those required for insulation, and we asked whether BEAF might control the deposition of epigenetic marks, as shown for other types of insulators [7,35,36]. We tested this possibility by measuring the levels of histone H3 methylated on lysine 9 (H3K9me3), a characteristic mark of heterochromatin, as a function of BEAF depletion. The deposition of H3K9me3 was strongly increased upon BEAF depletion (Figure 6A). Double immunostaining analysis showed that this increase was specific, as RNA polymerase II, actin, or unmodified histone H3 levels were unchanged (Figure 6A and 6B, Figure S3A and S3B). Numerous and broader H3K9me3 foci not restricted to heterochromatin regions appeared in BEAF-depleted cells (Figure 6B, 3× panels; [37]), strengthening the view that H3K9me3 also acts to influence gene expression in euchromatin [8,38,39]. ChIP-on-chip analysis confirmed that discrete H3K9me3 peaks are found in many promoter regions, including those associated with a dual-core (Figure S3C). However, these H3K9me3 peaks appear to be distinct from the broader and larger H3K9me3 peaks found in regions where genes are known to be repressed (e.g., *eye*, Figure S3C) and where the methylK27 mark is also present (not shown; B. Schuettengruber unpublished data).

We further tested if BEAF affects the deposition of H3K9me3 marks into dual-cores by performing ChIP analysis using anti-H3K9me3 antibodies on BEAF-depleted, DREF-depleted, or control cells (Figure 6C). BEAF-depletion led to a significant and reproducible increase of approximately 8-fold in H3K9me3 levels for the dual-cores linked to *snf-cdk7*, similar to that obtained for *mei-S332* and *CG1430*, and in stark contrast to the stable levels found for the *actin* control (Figure 6C; unpublished data). In contrast, no variation in H3K9me3 levels could be found upon DREF depletion (Figure 6C), showing that this increase is specific to BEAF depletion. This result also rules out that the changes we observe overall could be due to off-target effects. Moreover, CDK7 depletion, which severely impaired cell-cycle progression (unpublished data), did not affect the levels of H3K9me3 (Figure 6C), indicating that their increase is not due to an indirect perturbation of the cell cycle upon BEAF depletion. Finally, H3K9me3 levels did not vary in control regions located a few kbp away from the dual-core, suggesting that BEAF controls the deposition of this mark locally (unpublished data). These results show that BEAF dual-cores are involved in blocking the deposition of H3K9me3 marks, fully consistent with their ability to positively regulate the expression of dual core-associated genes.

### BEAF Positively Regulates Gene Expression by Restricting the Deposition of H3K9me3

**Figure 7:**
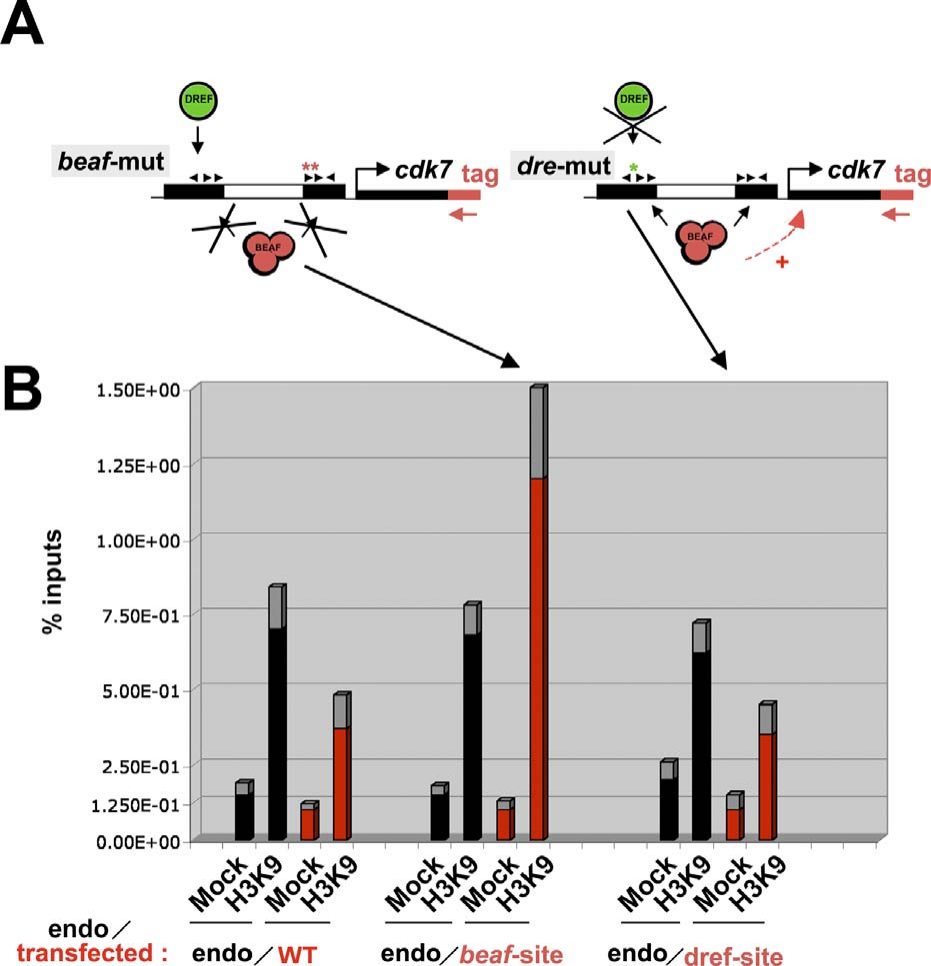
BEAF—But Not DREF—Binding Sites Restrict the Deposition of H3K9me3 Marks in Dual-Cores (A) Schematic representation of Dcore_38D mutated for its DREF (*dre-mut*; see Figure 5) or BEAF (*beaf-mut*) binding sites. The mutated CGATAs (arrowheads) are represented by the *. (B) Quantitative PCR analysis following ChIP with mock control or with anti-H3K9me3 antibodies of both transfected wild-type (WT) or mutated constructs (A) and endogenous ("endo") Dcore_38D from the same batch of cells. The *y*-axis shows the percentage of material precipitated from the inputs. For transfected constructs, the percentage was normalized to the DNA copy-number in the input (for details, see Materials and Methods). Experimental error is denoted by the differentially colored (gray) portion at the top of each bar.

To confirm that the observed increase in H3K9me3 levels is directly linked to the activity of BEAF, we introduced mutations in two of the CGATA motifs of the dual-core associated with *cdk7* (“*beaf-mut*”, Figure 7A) and transfected this construct or constructs harboring a wild-type dual-core or a dual-core mutated in the DREF site (*dre* mutant, Figure 7A) into cells. Quantitative PCR analysis of chromatin immunoprecipitated with anti-H3K9me3 antibodies showed that mutation of the BEAF site led to an increase in H3K9me3 levels of approximately 3.8-fold compared to wild-type or *dre* mutant constructs (Figure 7B), establishing that BEAF directly controls the deposition of H3K9me3. This did not affect the levels of H3K9me3 in the endogenous *cdk7* dual-core, as measured from the same batch of transfected cells, showing that the observed increase is indeed specific for the mutated dual-core. We conclude that BEAF serves to restrict the deposition of H3K9me3 marks into dual-cores.

**Figure 8:**
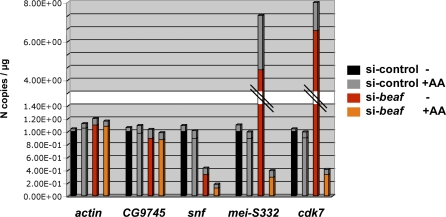
BEAF Dual-Cores Protect Genes from Chromatin Repression Quantitative RT-PCR analysis of the expression of—from left to right—*actin*, *CG9745*, *snf*, *cdk7*, and *mei-S332* in control cells (black and grey bars, in the absence or presence of 5 μM AA, respectively) or BEAF-depleted cells (red and orange bars, in the absence or presence of 5 μM AA, respectively). The *y*-axis shows the number of copies of amplification products per μg of RNA which was normalized for each gene in control cells, where N = 1 corresponds to 11,200 copies of *actin*, 12,900 copies of *CG9745*, 75,430 copies of *snf*, 64,500 copies of *mei-S332*, and 129,800 copies of *cdk7*. Experimental error is denoted by the differentially colored portion (gray) at the top of each bar.

The deposition of epigenetic marks is critical for regulating gene activity at the level of chromatin accessibility [9,12,13], which may account for the positive effect of BEAF on gene expression. We sought to determine whether BEAF-regulated deposition of H3K9me3 marks affects the expression of cell-cycle genes. BEAF-depleted or control cells were treated with anacardic acid (AA), a histone acetyltransferase (HAT) inhibitor that globally affects gene expression by altering the accessibility of chromatin [40]. AA treatment did not affect the expression of either control genes or dual core-associated genes (compare grey and black bars in Figure 8). In contrast, AA severely compromised the activation of *snf*, *cdk7*, or *mei-S332* upon BEAF depletion compared to untreated BEAF-depleted cells (Figure 8; unpublished data). This result strongly supports a model whereby BEAF restricts the deposition of methylated H3K9 marks, thereby protecting the expression of dual core-associated genes from repression by chromatin (see Discussion).

### Regulation of Gene Expression Involves the Cooperative Binding of BEAF to Dual-Cores

**Figure 9:**
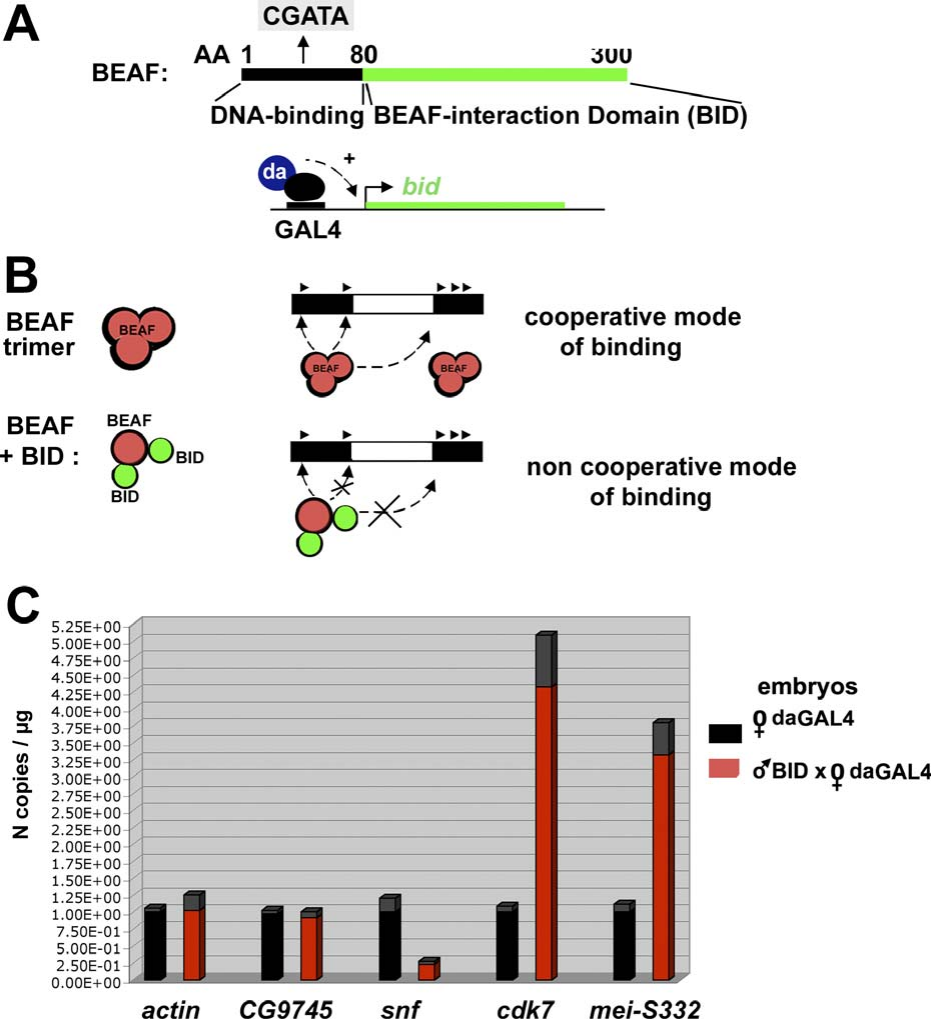
Regulated Gene Expression Requires Cooperative Binding of BEAF to Dual-Cores and Is Impaired by BID (A) Schematic representation of BEAF. Its N-terminus is the DNA binding domain (AA 1–80) which targets one CGATA motif [17,23–25]. Its C-terminus, required for assembly of BEAF complexes, is called the BEAF self-interaction domain (BID; AA 80–280). As a result, expression of BID under the control of the GAL4-daugtherlesss (da) activator in transgenic flies dominantly competes with the cooperative binding of BEAF [25], as represented in (B). (B) Schematic representation showing how the cooperative binding of BEAF complexes to clustered CGATAs of dual-cores is compromised by expression of BID upon its assembly with BEAF subunits. (C) Quantitative RT-PCR analysis in 4–8-hour embryos expressing a GAL4-driven BID transgene. The results are expressed as copy number of amplification products measured for each gene in embryos expressing BID or in embryos expressing the da-GAL4 driver alone. The *y*-axis shows the number of copies of amplification products per μg of RNA normalized for each gene in control cells, where N =1 corresponds to 9,900 copies of *actin*, 11,200 copies of *CG9745*, 77,300 copies of *snf*, 59,400 copies of *mei-S332*, and 116,200 copies of *cdk7*. Experimental error is denoted by the differentially colored (gray) portion at the top of each bar. Note that BID expression leads to lethality at later stages of development [25].

Are these variations in gene expression related to the cooperative binding of BEAF to the two clusters of CGATAs present in dual-cores? We sought to answer this question by using transgenic fly lines expressing the C-terminal BEAF self-interaction domain (BID in Figure 9A) under the control of a GAL4 activator. BID lacks the BEAF DNA-binding domain, impairing the insulating activity of BEAF [25] by preventing its cooperative binding to two nearby CGATA clusters (Figure 9B). Importantly, defects in expression of *cdk7*, *snf*, and/or *mei-S332* were highly similar in embryos expressing BID to that observed in BEAF-depleted cells (compare Figures 9C and 4B). This result supports our conclusion that BEAF binding is required to regulate these genes in vivo. It also suggests that the cooperative binding of BEAF to conserved *dual-cores*, which is abolished by BID, may be important for the regulation of gene expression by BEAF. Accordingly, cell functions enriched in BEAF dual-cores include GOs (Table S1) that correspond to phenotypes observed following expression of *beaf* mutants, which are lethal to flies [25], or to GOs found to genetically interact with these mutants [28].

## Discussion

Results of our in silico analysis reveal ∼1,720 BEAF dual-cores in the Drosophila melanogaster genome that share a striking organization (Figure 1E). Genome-wide ChIP-on-chip analysis detects approximately 1,800 significant BEAF binding sites (C. M. Hart, unpublished observations), suggesting that our dual-core database encompasses most in vivo BEAF binding sites. The few (<100) additional peaks not included in our database but detected by ChIP-on-chip analysis may correspond to elements initially scored as single elements but whose organization is close to that of dual-cores. These rare exceptions are in part due to the computer stringency of the dual-core signature. For example, BEAF-1255 can be bound by BEAF in vivo (Figure S4), yet this element could not be scored as a dual-core because one out of five of its clustered CGATA motifs lies 3 bp outside the defined 100-bp window (‘out' in Figure S4). Furthermore, approximately 10% of the minor BEAF sites are found in regions lacking any CGATA motifs, including the *scs* insulator (unpublished data) [16]. Since this region is not directly bound by BEAF, it is thus possible that some of the minor BEAF peaks are due to indirect interactions between BEAF and other insulator proteins, as previously suggested for the *scs′*–*scs* pair of insulators [16]. Other protein–protein interactions that regulate BEAF binding could also involve the splicing variant of the *beaf* gene itself, called BEAF-32A [23], which does not harbor the BEAF DNA-binding domain that recognizes clustered CGATA motifs. ChIP-on-chip analysis using antibodies that also recognize this isoform showed no additional major peaks (Figure S5, compare ‘−32A' with ‘+32A'), indicating that dual-cores constitute the main binding sites for both BEAF isoforms. Finally, we note that the BEAF-32A isoform is unlikely to play a major role in the activities described here, as its binding is dispensable for the insulating function of BEAF [20], and its expression is not essential for the development of embryos into adult flies [29]. Taken together, our results show that the BEAF dual-core signature is a bona fide mark that identifies a *cis*-regulatory element that regulates the expression of nearby genes.

Results of our experiments using both BEAF depletion in tissue culture cells and BID expression in vivo provide clear evidence for specific functions of the BEAF dual-cores, reflected by a selective association with genes that control cell-cycle and/or chromosome organization/segregation. The competition between DREF and BEAF for binding to nested consensus sequences is also supported by ChIP analyses showing that DREF targets' identical sites [34] clearly enriched nearby genes associated with the cell cycle and chromosome dynamic GOs (Figure S6; unpublished data). Thus, while DREF levels increase at the G1/S transition to activate *mei-S332* and *cdk7* within the appropriate window for cell-cycle progression [30–32], BEAF may further facilitate this activation by restricting the deposition of H3K9me marks. Indeed, over-expressing BEAF was shown to reduce the phenotypes related to cell-cycle progression in flies that over-express DREF [33], supporting a role for BEAF in controlling the cell cycle. Such a model is also supported by our observation that AA treatment strongly represses these genes in BEAF-depleted cells and that mutation of the BEAF-binding site in a dual-core results in a local increase in H3K9m3 levels. In addition, computer analysis of micro-array expression data for *Drosophila* embryos during early development shows that the 545 genes associated with dual-cores are positively correlated with *beaf* expression (Figure S7A), in contrast to genes unlinked to these elements (*p*-value ∼ 3e-17 according to the Kolmogorov-Smirnov test). This strict correlation further indicates that BEAF has a global positive role on gene expression genome-wide, and similar analyses did not reveal any significant correlation change between genes whose TSS is closely juxtaposed (<100 bp) to dual-cores, including *snf* or *cdk7* (Figure S7B), compared to genes whose TSS is more distant (500 bp). Accordingly, the cell-cycle and chromosome dynamics GOs that include *cdk7* and *mei-S332* are enriched for positively correlated genes (see our database for a detailed list). Taken together, our results show that BEAF could play an important role in chromosome organization during the cell cycle through a regulated switch involving the BEAF–DREF competition: According to such a mechanism, BEAF would restrict the deposition of H3K9me3, allowing dual-core–associated genes to remain in a potentially active state, while controlling the time of activation of cell-cycle GOs by DREF. Accordingly, BEAF depletion leads to down-regulation of genes associated with a dual-core lacking a DREF element (CG10946, *ras*, CG1430, *Janus*, CG1444), but to increased expression of CG32676, *mei-S332*, *cdk7*, CG10944, and *ser*, which are under the control of DREF-associated dual-cores (Figure 4). In the latter case, the apparent contradiction between the positive—restriction of H3K9me3 deposition—and negative effects of BEAF can be reconciled by our results showing that BEAF controls the activation of these genes by DREF. BEAF depletion relieves the competition for binding by DREF, leading to the increased expression of *cdk7* or *mei-S3332* in spite of an increased deposition of H3K9me3 marks under these conditions. Mutating the DREF or BEAF binding sites of DREF-associated dual-cores (Figures 5 and 7) allows for distinguishing between these different effects on the expression of linked genes.

It is intriguing that the spacers of dual-cores are well-conserved. One possibility is that they may be preferentially bound by a nucleosome, as recently shown for CTCF insulators [41]. Supporting this idea, the known dual core-spacers correspond to nuclease-resistant “cores”, between two nuclease-hypersensitive sites (BE76, *scs′*) [20,24,26] (Figure S8), where a nucleosome may be present (C. M. Hart, unpublished observations). Indeed, we found that dual core-spacers fall within predicted nucleosome-positioning sequence (NPS) databases [42–44], as indicated by NPS/dual-core sequence alignments (Figure S8; not shown), possibly accounting for the conserved organization of dual-cores. Our results further suggest that the cooperative binding of BEAF across these AT-rich spacers may be important for BEAF function. Indeed, expression of BID, which prevents its cooperative binding across the spacers, mimics the effect of BEAF depletion on the expression of dual-core–associated genes, as also found by mutagenesis of two CGATA motifs from one dual-core cluster. However, BEAF still efficiently binds in vivo to the few dual-cores that harbor a shorter spacer (<150 bp; e.g., see Dual-core 1,254, Figure 1; unpublished data), indicating that the conserved dual-core–spacer is dispensable for BEAF binding. Recent reports have shown that gene expression is differentially regulated through nucleosome positioning in several species [12,13,42,43]. Positioned nucleosomes may restrict promoter accessibility in yeast, and pausing of RNA polymerase II facing the +1 nucleosome may be regulated through nucleosome positioning in *Drosophila* [44]. Similarly, dual-cores are also closely associated with TSSs, and a potential link to nucleosome positioning strengthens the view that BEAF may regulate chromatin accessibility for gene expression through a restriction of the deposition of methylated H3K9 marks into dual-cores.

Our model whereby dual-cores regulate the deposition of specific epigenetic marks is in agreement with the activity of other known insulators [6,7,9–11]. Variations in H3K9me3 levels might affect the interplay between the deposition of H3K9me3 and acetylated histone H4 (H4Ac) marks [45]. However, no variation in the deposition of H4Ac could be found in dual-cores compared to control regions after BEAF depletion (unpublished data). This is not surprising, as BEAF has no de-silencing activity on its own [5,25]. Computer analysis failed to reveal any enrichment of dual-cores near the 3′UTR of genes, and the activity of dual-cores may thus essentially play a role in regulating chromatin accessibility near promoter regions, but not within the 3′ border of genes. Furthermore, the insulating activity of BEAF was demonstrated in the context of two dual-cores bracketing a transgene [5,25], and most likely also involved higher-level chromatin organization [2]. Although not enriched near the 3′UTR of genes, dual-cores still bracket/separate groups of genes clustered within 5–15 Kbp, a genomic context that may further require insulating activity to block promiscuous enhancer–promoter interactions and involve DNA looping between distant insulators [2]. It has recently been shown for a Su(Hw) insulator that the regulation of gene expression may further depend on its genomic environment [46]. Also, other dual-cores are often found in the vicinity of genes exposed to repression by heterochromatin (see our genome-wide database), and the function of BEAF may be particularly important in this context [17,20,23,24]. We propose that the BEAF dual-cores closely linked to a restricted array of several hundred genes define a family of insulators that provide a link between chromatin organization and the cell cycle.

## Materials and Methods

### Bioinformatic analysis, availability of predictions, dual-core sequences.

All genome-wide predictions and analyses are available on our Web site: http://www.sfu.ca/~eemberly/insulator/. Additional information, including DNA sequences of single elements, dual-cores or dual-core–like elements, and their position relative to genes or other genomic features (GOs) can be directly retrieved from our Web site.

### Dual-core–like prediction, distribution of CGATA sites in dual-cores.

Each single BEAF element that was not a part of a dual-core element was analyzed for the presence of a “dual-core–like” signature. We define single elements as consisting of three CGATAs within 200 bp, and a dual-core–like element as a single BEAF element (three CGATAs) associated with a second nearby (<800 bp) cluster of two CGATA sites within a 100-bp window. 1,226 BEAF elements fit into this classification, including all previously identified dual-cores (BE76, BE28, BE51, Jan/Ser(BE83)). The position of each CGATA site within a dual-core sequence was analyzed relative to the position of the rightmost site of the first BEAF single element. In Figure S1, the position of each CGATA motif was measured from the average position (taken as position 0 on the *x*-axis) of all the CGATA locations in the first BEAF single element of the dual-core. This removes any ambiguity in defining the starting position of the sequence, allowing more precise mapping of dual-cores with respect to gene promoters.

### Statistical significance of dual-cores.

We predicted dual-cores by pairing together the genome-wide set of 7,045 single BEAF elements that were separated by a spacer <L bp. The statistical significance of the number of predicted dual-cores as a function of spacer length L was assessed by comparing it to the expected number for randomly spaced elements. The *p*-value was found to reach a flat minima for 600 bp < L < 3,000 bp. For larger L values, the predictions decreased in significance, eventually becoming no more significant than chance. There are 1,720 dual-cores, L = 800 bp with a *p*-value of 1e-9, in the sequenced Drosophila melanogaster genome.

### Statistical significance of promoter distances to dual-cores.

The statistical significance of the number of dual-cores within +/− d bp of a promoter was assessed by comparing it to the number expected for randomly placed elements. Out of 1,720 dual-core elements, 545 fall within +/− 500 bp of a promoter. Beyond this distance, the *p*-value was found to decrease in statistical significance, yet 850 dual-cores reside within 2,000 bp of a promoter. Additional dual-cores are found close to genes or groups of genes (see our database).

### Statistical significance of the distribution of BEAF dual-cores.

In order to analyze the distribution of dual-cores, we calculated the statistical significance for a minimum number of dual-cores, 2, 3,…x dual-cores (DC) to be found along 5, 10, …100 kbp of DNA (W). For a given W and DC, we predicted N(W,DC), the frequency of dual-cores for a certain DNA length. To assess the significance of N(W,DC), we compared it to the number of randomly distributed elements for the same DNA length. If the probability of a random dual-core element to occur within a window of size W is p, then the probability that there are ≥DC elements in W is P(W,DC) = B(x > DC,W,p), where B is the binomial distribution. The expected number of domain predictions for these random elements is then E(W,DC) = Nwin(W)*P(W,DC), where Nwin(W) is the number of non-overlapping windows of size W in the entire genome. The *p*-value for N(W,DC) can then be evaluated using the expected number E(W,DC) as a function of W and DC. We find W = 10 kb and DC = 2 to yield the statistically most significant BEAF dual-core distribution in pairs (*p*-value ∼ 1.01e-33).

### GO analysis.

The statistical significance of a GO class was assessed using the binomial distribution, *p*-value = B(x, N, p), where x is the number of genes within the given GO class in a set of N predicted genes, and p is the probability of that GO class in the entire annotation. See our database for a complete listing of all GO analyses of positively correlated genes with or without BEAF dual-cores or DREF elements in their promoters.

### Genomic expression analysis and microarray data

Genome-wide *Drosophila* gene expression data (Figure S7) covering the first 12 hours of embryonic development are available from the Berkeley *Drosophila* Genome Project. Twelve time points were collected, each with three replicates. Each gene g in the genome has an expression profile containing 12 data points (g_i_ = (x_1_, x_2_, …, x_12_)). For a given pair of genes, we calculated the Pearson correlation coefficient between their respective expression profiles. We then calculated the correlation coefficient between a given set of genes and a given reference gene. To test whether two sets of genes had statistically different correlation coefficient profiles, we used the Kolmogorov-Smirnov test, which assigns a *p*-value to the likelihood that two samples of a continuous random variable come from the same parent distribution.

### Chromatin immunoprecipitation of BEAF, H3K9me3.

Chromatin immunoprecipitation (ChIP) was done according to the Upstate protocol using control or *beaf* siRNA-treated cells. Equivalent amounts of chromatin samples were sonicated using a Diagenode Bioruptor and immunoprecipitated with 4 μl of anti-H3K9me3 (Abcam). Precipitated DNA was analyzed by real-time PCR in parallel with genomic DNA using a Roche Light Cycler and a Light Cycler FastStart DNA Master SYBR green kit. The amplified DNA fragments (<250 bp) cover regions corresponding to the indicated elements (Figures 6 and 7). ChIP with anti-BEAF was performed as previously described [34] with 10 μl affinity-purified anti-BEAF antibodies that recognize (Figure S5) or not (Figure 2) the BEAF-32A isoform or IgG. The immunoprecipitated DNA was analyzed in parallel with input genomic DNA as a standard. For ChIP-on-chip assays using H3K9 antibodies, precipitated DNA was amplified by ligation-mediated PCR (LM-PCR). 4μg of each amplified sample was used to hybridize on 3 × 385 K tiling microarrays representing the euchromatic, non-repetitive regions of the Drosophila melanogaster genome sequence (Flybase release 4.3) from Nimblegen Systems (GEO accessions: GPL3352, GPL3353, GPL3354). To calculate whether the levels of enrichment are statistically significant for each array, a normal distribution was calculated, with the assumption that the mode and median absolute deviation of the normalized log2 ratios are the average and the standard deviation of the normal distribution, respectively. Assuming that the normal distribution covers the entire background noise (non-significant signals), a *p*-value was calculated for each oligonucleotide signal. For the two replicate samples of each profile, each pair of probe *p*-values were then combined using a Chi Square law with 4 degrees of freedom. Finally, correction for multiple testing [47] was applied to the combined *p*-values. Only oligonucleotides with final *p*-values (for combined replicates) < 1E-04 were considered to be significantly enriched for the signal.

### Expression analyses, siRNA treatments, transfections, expression of *beaf* mutants in embryos.

For siRNA treatments, exponentially growing *Drosophila* Schneider SL2 cells were maintained between 1 and 4 × 10^6^ cells/ml in Schneider's *Drosophila* medium (SDM, GIBCO, Invitrogen) supplemented with 10% Fetal Bovine Serum (FBS, Sigma) and 1% penicillin/streptomycin (GIBCO, Invitrogen). Cells were diluted to a final concentration of 1 × 10^6^ cells/ml in SDM without FBS, and 400 μl of 2 μM *beaf*32, *dref* or *cdk7* double-stranded RNAs (dsRNA) were added directly to 10 ml of cells which were then plated on 75-cm^2^ T-flasks (Sarstedt), immediately followed by vigorous agitation. dsRNAs were synthesized using full-length cDNAs of the above genes as templates. Primers consisted of a complementary template portion, a floating end with a T7 promoter and an EcoR1 site located at the other end. 5 μg of DNA template were transcribed for 2 hours at 37 °C in the presence of 0.5 mM rNTPs, 10 mM DTT, 120 units RNAse inhibitor, 60 units T7 polymerase in its 1× buffer in a 100 μl final volume. cDNA degradation was performed for 30 to 40 minutes at 37 °C in the presence of 4 units RQDNase in a 400 μl final volume of the recommended buffer. dsDNAs were then extracted with phenol/chloroform, ethanol-precipitated, and solubilized in 20 μl TE, pH 7.5. The resulting sequences were checked for potential off-target effects by performing searches with dsCheck [48] (http://dsCheck.RNAi.jp/). Treated cells were incubated for 2 hours at 25°C, followed by addition of 20 ml of SDM containing FBS, and cells were incubated for an additional 5 days. Depletion of *beaf*32 mRNA was assayed by RT-PCR at 1, 3, or 5 days after treatment. Cells were grown for 5–6 days, and samples were recovered for total RNA, immunostaining, or immunoblotting analysis. FACS analyses were performed after resuspending control or BEAF-depleted cells and staining their DNA with propidium iodide. Analysis of gene expression was performed by quantitative RT-PCR on cDNAs prepared by RT-PCR from BEAF-depleted or control cells (+5–6 days), untreated or treated with AA (5 μM) for 24 hours. Each measurement was reproduced three times and in two independent RNA extraction experiments. For gene expression analysis, cDNAs prepared from control or BEAF-depleted cells were quantified in parallel with genomic DNA by RT-PCR using a Qiagen Light Cycler. Transfections of plasmids were performed using Lipofectamine (Invitrogen) for 2 hours according to the manufacturer's instructions, 48 hours before RNA purification. Measurements of gene expression for the transfected (wild-type or mutant) constructs were performed using primers that specifically amplify cDNAs from the tags introduced at the 5′ and 3′ borders (see Figure 5) and that were unable to amplify cDNAs from untransfected cells (unpublished data). Expression was normalized to the copy number of transfected constructs estimated by quantitative PCR of input genomic DNA. For endogenous genes, the primer sequences were selected from the coding regions (≈1,000 bp 3′ from promoter start) of each gene. For endogenous *cdk7/snf*, the selected primers lie outside (15 bp 5′ or 3′) of the tags. For other analyses, two primer pairs were used alone or in combination to confirm the specific increase/decrease in gene expression, using actin as a control. For quantitative RT-PCR analysis in embryos, males with the BID transgene on Chromosome 2 (CyO/Sp1; BID2B) were crossed with virgin females harboring an embryonic da-GAL4 driver (*daughterless*) on Chromosome 3. The corresponding measurements were compared to those from embryos expressing da-GAL4 alone or from BID2B embryos without a da-GAL4 driver.

### Mutagenesis of dual-cores.

For mutagenesis of Dcore38_D, a genomic DNA fragment harboring the first exons of *cdk7* and *snf* was cloned, and PCR-mediated mutagenesis was performed using primers that contain mismatches as followed: the *dre* (DREF site) mutant sequence is TAgCGATA and disrupts DREF binding but preserves the CGATA consensus of BEAF. The BEAF site mutant was produced by mutagenesis of two of the CGATA consensus in one cluster of the dual-core, using the *tt*ATA mismatches critical for BEAF binding [17,23–25].

### Polytene chromosomes, immunostaining analyses, Western blotting, and mapping of nuclease-sensitive sites.

Immunostaining analyses were performed using affinity-purified mouse or rabbit anti-BEAF-32B (1:100) as previously described [34,49], using the indicated affinity-purified antibodies or commercially available anti-acetyl-Histone H4, anti-H3K9me3, anti-H3, anti-RNA polymerase II (Upstate), or anti-actin antibodies (Sigma). Double immunostaining of siRNA-treated cells was performed in duplicates and in parallel for control or BEAF-depleted cells treated for 1, 3, or 5 days. Each experiment was repeated three times. DNA was stained with 500 ng/ml DAPI or 1 μg/ml Hoechst, and coverslips were mounted with 4 μl of antifading mix and sealed with nail polish. Slides with siRNA control or BEAF-depleted cells were analyzed using the same acquisition parameters using a Leica DMRA2 microscope. Mapping of BEAF dual-cores and immunolocalization of anti-BEAF signals was performed over >10 Mbp for Chromosome 2 and X chromosome, showing striking correspondence (analysis available upon request). For mapping of nucleases-sensitive sites (Figure S8), freshly isolated nuclei from approximately 10^8^ cells were digested with very low concentrations of either microccocal nucleases or DNAase I essentially as previously described [17,20,23,24], and the purified DNA was further digested with *Pvu*II and run onto a 1.2% agarose gel for Southern blotting. Naked DNA controls were similarly digested. A *Pvu*II-*Eco*RI end-labeled DNA fragment was used to probe specifically the region containing the dual-core region. Western blotting was performed using anti-actin or anti-BEAF antibodies. As a control, genomic DNA was first purified and then digested with MNase and Pvu II (+/− EcoRI to mark the 5′ border of the dual-core) before analysis by Southern blotting. Western blotting was performed as previously described [17,24] using anti-actin, anti-H3K9me3, anti-mei-S332, or anti-BEAF antibodies.

## Supporting Information

Figure S1 Statistical Analysis of Dual-Cores (A,B) Plots showing the distribution of all 12,058 CGATA motifs from dual-cores (A) and the locations of their AT-rich spacers (B) as in Figure 1C and 1D, except that positions were calculated according to average positions of the three CGATAs in the first (left) cluster to define position zero.
(C) CGATA motifs in the second cluster are enriched near the border of the spacer (+200–300 bp), while fewer localize at larger distances.
(89 KB PDF)

Figure S2 Depletion of BEAF Impairs Protein Levels of Key Cell-Cycle Factors Immunoblotting experiment showing the protein levels of BEAF, MEI-S332, and CDK7 compared to loading controls (ACTIN, DSP1), after siRNA-mediated depletion of BEAF or control treatment. 1.0, 3.0: standard, or 3-fold excess protein loading, respectively.
(43 KB PDF)

Figure S3 BEAF Controls the Levels of H3K9me3 Marks (A,B) Immunostaining analysis using (A) anti-histone H3 (green) and anti-actin (red) antibodies or (B) anti-H3K9me3 antibodies, in SL2 control (“control”) or BEAF-depleted (“*beaf*”) cells. Enlargements of confocal images after staining with anti-H3K9me3 antibodies are also shown (3×). DNA was counterstained with Hoechst. Bar, 10μm. (C) Profile of H3K9me3 and position of BEAF Dcores on the X chromosome corresponding to the Xdcore_38D region (first dual-core from right) or to the *eye* locus from ChIP-on-chip data. Note that promoter regions often fit into discrete H3K9me3 peaks distinct from the major H3K9me3 peaks of repressed loci (e.g., *eye*) that are also enriched for the H3 methylK27 mark (see text).
(156 KB PDF)

Figure S4 BEAF Elements Resembling Dual-Cores Are also Bound by BEAF In Vivo The figure shows one of the exceptions for a region where some BEAF binding is detected (graph in green) by genome-wide ChIP-on-chip analysis (approximately 1,800 peaks total) yet which is not included in our database of dual-cores (1,720 dual-cores). This region was not scored in the dual-core database because the second CGATA in the first cluster is 103 bp away (‘out') instead of the defined window of 100 bp. TSSs and primary transcript are depicted on the top graphs (see purple bars and blue line, respectively).
(474 KB TIF)

Figure S5 The BEAF-32A Splicing Variant also Binds to Dual-Cores The panel shows an alignment of ChIP-on-chip analysis (graphs in green) using anti-BEAF antibodies that recognize the BEAF-32A splicing variant (‘+32A') or not (−32A). The red bars mark the position of significant peaks over the same region of the X chromosome (nucleotide positions 4,950,000 to 5,300,000) as shown in Figure 2. TSS (blue bars) and primary transcripts (purple lines) are shown on top.
(1.4 MB TIF)

Figure S6 Respective Enrichment of BEAF Dual-Cores and DREF Elements for Several Gene-Class Ontologies *p*-Values for gene annotations (GOs) of BEAF dual-cores-only (“dual-cores-only”) versus dual-cores containing additional TATCGATA consensus sites for DREF (“dual-cores-DREF”) [50]. The ratio of *p*-values is shown for each independent GO category and highlights a greater enrichment for BEAF dual-cores–only sites in chromosome organization (left) and for dual-cores–DREF sites in cell-cycle and apoptosis (right). DREF competes with BEAF for binding to a nested consensus sequence [34] present in dual-cores marked by a “_D” sign (see our Web site). These are significantly enriched in common GOs, including cell-cycle, in agreement with genetic interactions between *beaf* and *dref* [33,50] (see text for details).
(124 KB PDF)

Figure S7 Genome-Wide Analysis of the Impact of BEAF Dual Cores on Transcription (A) BEAF dual-cores have a global positive impact on transcription. Distribution of correlation coefficients between the expression profile of genes with (red) or without (black) BEAF dual-cores (i) in their promoters (see Materials and Methods). “+” and “−” signs indicate statistical enrichment for co-regulated and anti-correlated gene expression profiles, respectively. As a positive control, the target genes for DREF [50] are enriched, as expected, for a minor subpopulation highly co-expressed with DREF (ii), but less significantly (*p*-value of 0.004 according to the Kolmogorov-Smirnov test) than BEAF, which has a more global positive effect on gene expression (*p*-value ∼ 3e-17 according to the Kolmogorov-Smirnov test).
(B) Distribution of the BEAF (CGATA, green boxes) and DREF (TATCGATA, red) motifs in the Dual-core 38_D with respect to *snf* and *cdk7* (TSS corresponds to the first colored bp).
(309 KB PDF)

Figure S8 The Spacers of BEAF Dual-Cores Fit into Nucleosome-Positioning Sequences (A,B) Relative positioning of CGATA BEAF consensus binding motifs and the position of putative NPSs predicted by submitting dual-core sequences to available databases [42,43] in the *cdk7* and *mei-S332* promoter regions (A) as well as in >20 cell-cycle regulatory genes (B) (see our Web site for a list). Predicted NPSs are indicated by purple boxes below dual-cores (A) or as an overlay of predicted NPSs (B). The relative position of nuclease-resistant cores is indicated (N; according to experiments as shown in (C)). These predictions fit with the positions of AT-rich dual-core spacers (see Figure 1D).
(C) Mapping of the accessibility of naked DNA control (top photograph) and of chromatin by nuclease digestion of nuclei (MNase,“M”; or DNAase I, “D”; see Materials and Methods). To map nuclease-resistant/sensitive regions with respect to CGATA clusters of dual-cores, purified genomic DNA was further digested with a second enzyme (*Pvu*II +*Not*I or *Eco*RI) which cuts into the first CGATA cluster or 50 bp 3′ of the second CGATA cluster, respectively (see dotted lines below the autoradiogram). The dual-core spacer fits into a nuclease-resistant core region bracketed by hypersensitive sites. Note that these features are not found in the naked DNA control, where genomic DNA was first purified before MNase digestion.
(239 KB PDF)

Table S1 Gene-Class Ontologies Associated with BEAF Dual-Cores GO terms for 1,720 BEAF dual-core target genes, which contain a dual-core within +/− 1,000 bp of their promoter.671 dual-core elements hit one promoter in the genome. The second column gives the number of annotated genes in that GO class, the third column gives the number of genes in dual-core/promoter sets in that GO class, the fourth column shows the expected number of genes in the predicted set given the observed class frequency. The corresponding *p*-value is given in the fifth column. GO terms have been binned into larger categories. Low-scoring GO classes underrepresented in the set of dual-core target genes are shown at the bottom. See our database for a complete listing and additional GO analysis.
(25 KB DOC)

## Abbreviations

AA: anacardic acid
BE: boundary element
BEAF: boundary element–associated factor
cdk: cyclin-dependent kinases
GOs: gene-class ontologies
H3K9me3: histone H3 tri-methylated on lysine 9
HAT: histone acetyltransferase
NPS: nucleosome-positioning sequence
Su(Hw): suppressor of Hairy-wing
TSS: transcriptional start site
