# Supplementary figures and images for: BEAF Regulates Cell-Cycle Genes through the Controlled Deposition of H3K9 Methylation Marks into Its Conserved Dual-Core Binding Sites
Source: PLoS Biol. 2008 Dec 23;6(12):e327. doi: 10.1371/journal.pbio.0060327 (PMC2605929; doi:10.1371/journal.pbio.0060327)

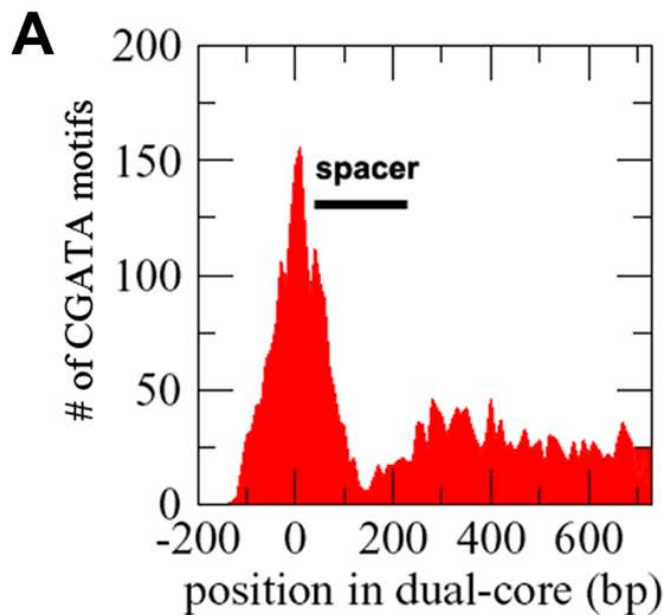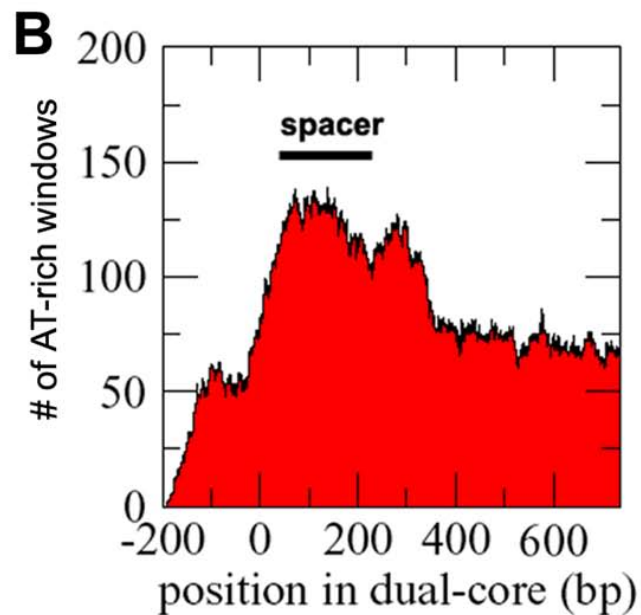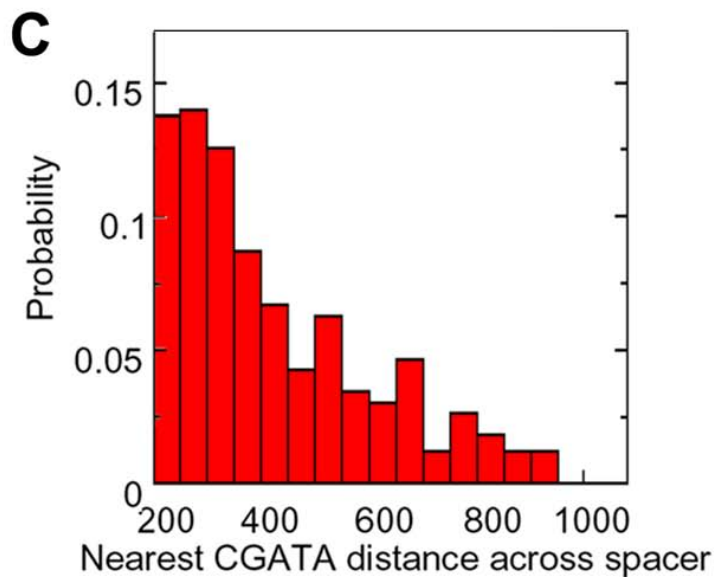

Supplement: Figure S1 — (A,B) Plots showing the distribution of all 12,058 CGATA motifs from dual-cores (A) and the locations of their AT-rich spacers (B) as in Figure 1C and 1D, except that positions were calculated according to average positions of the three CGATAs in the first (left) cluster to define position zero. (C) CGATA motifs in the second cluster are enriched near the border of the spacer (+200–300 bp), while fewer localize at larger distances. (89 KB PDF) [file pbio.0060327.sg001.pdf]

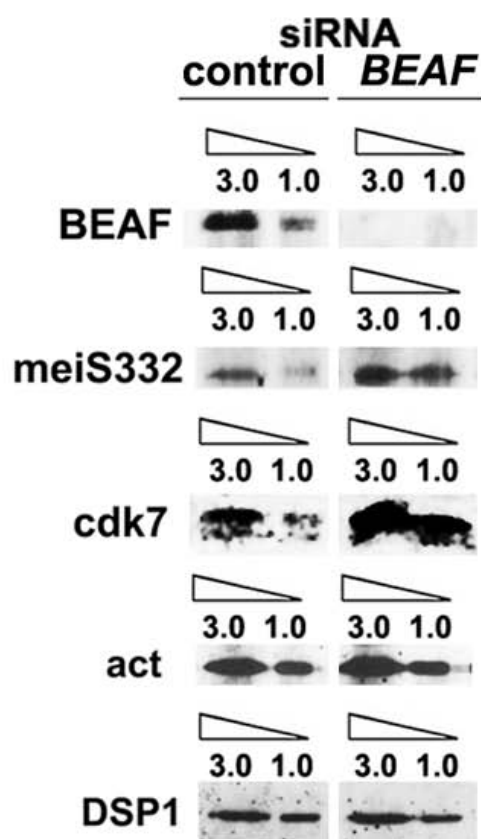

Supplement: Figure S2 — Immunoblotting experiment showing the protein levels of BEAF, MEI-S332, and CDK7 compared to loading controls (ACTIN, DSP1), after siRNA-mediated depletion of BEAF or control treatment. 1.0, 3.0: standard, or 3-fold excess protein loading, respectively. (43 KB PDF) [file pbio.0060327.sg002.pdf]

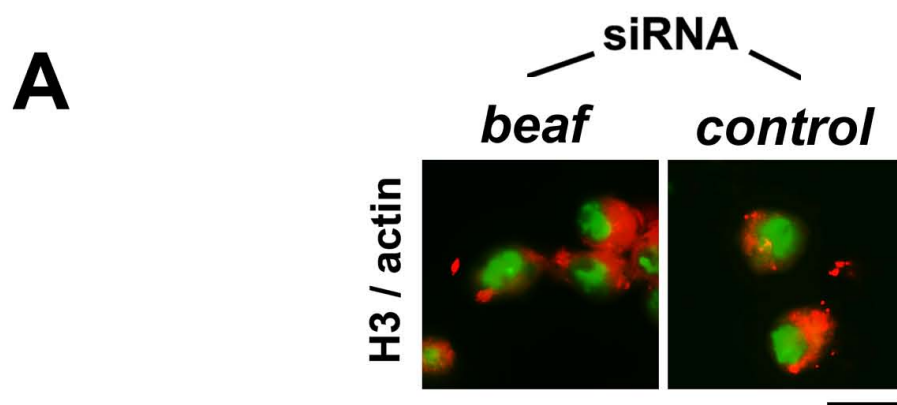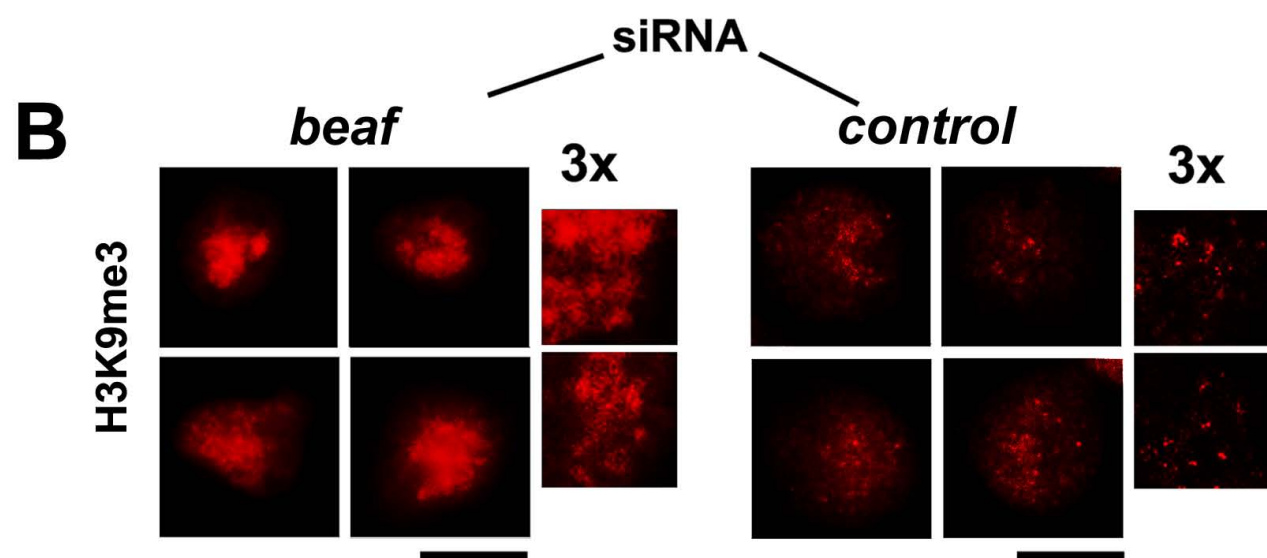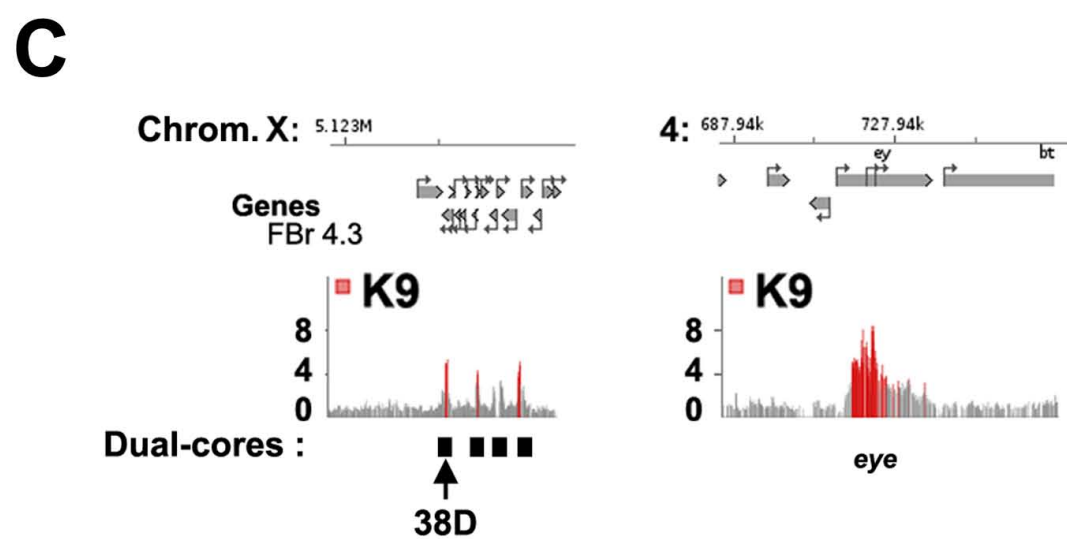

Supplement: Figure S3 — (C) Profile of H3K9me3 and position of BEAF Dcores on the X chromosome corresponding to the Xdcore_38D region (first dual-core from right) or to the eye locus from ChIP-on-chip data. Note that promoter regions often fit into discrete H3K9me3 peaks distinct from the major H3K9me3 peaks of repressed loci (e.g., eye) that are also enriched for the H3 methylK27 mark (see text). (156 KB PDF) [file pbio.0060327.sg003.pdf]

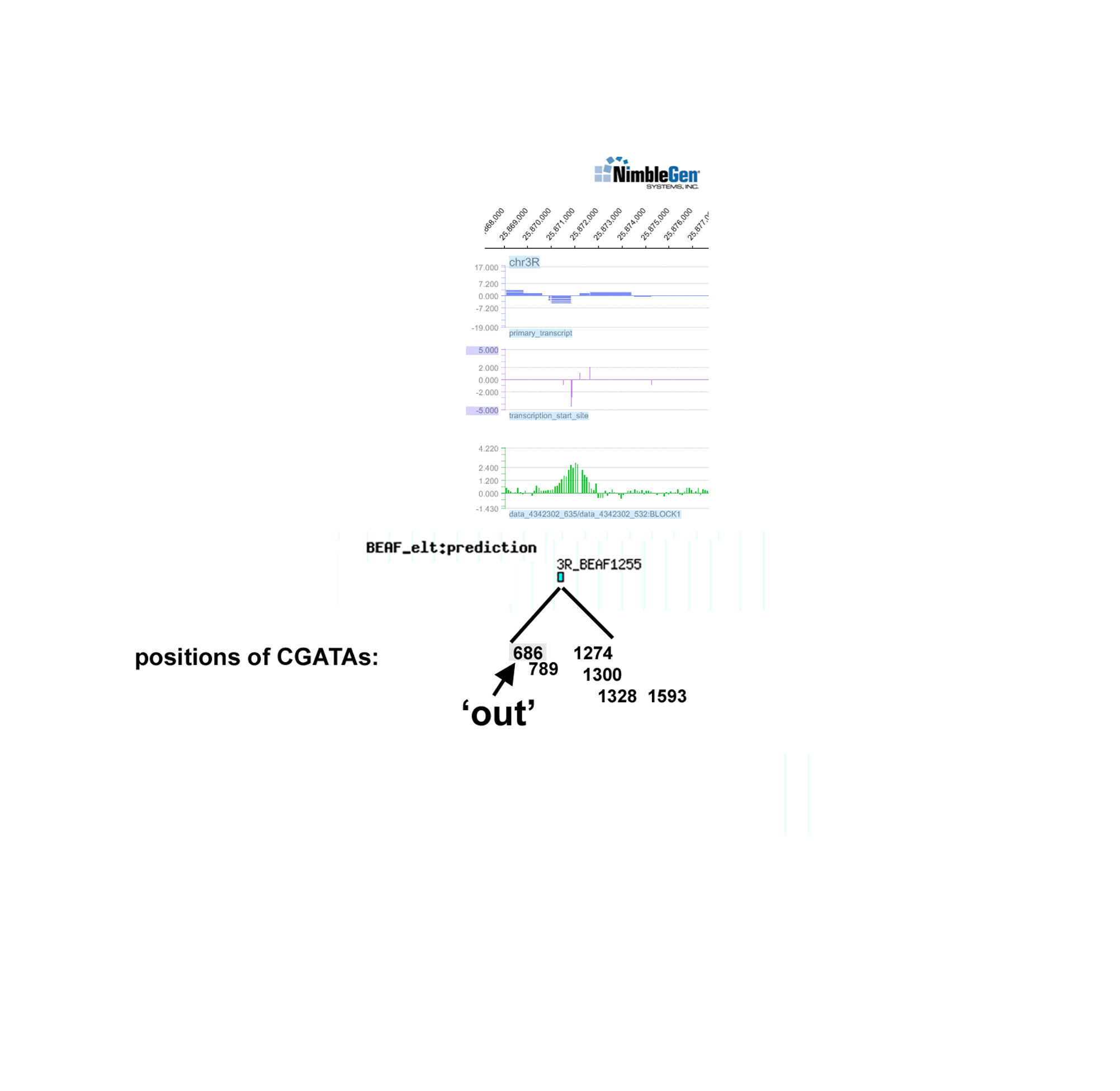

Supplement: Figure S4 — The figure shows one of the exceptions for a region where some BEAF binding is detected (graph in green) by genome-wide ChIP-on-chip analysis (approximately 1,800 peaks total) yet which is not included in our database of dual-cores (1,720 dual-cores). This region was not scored in the dual-core database because the second CGATA in the first cluster is 103 bp away (‘out') instead of the defined window of 100 bp. TSSs and primary transcript are depicted on the top graphs (see purple bars and blue line, respectively). (474 KB TIF) [file pbio.0060327.sg004.tif]

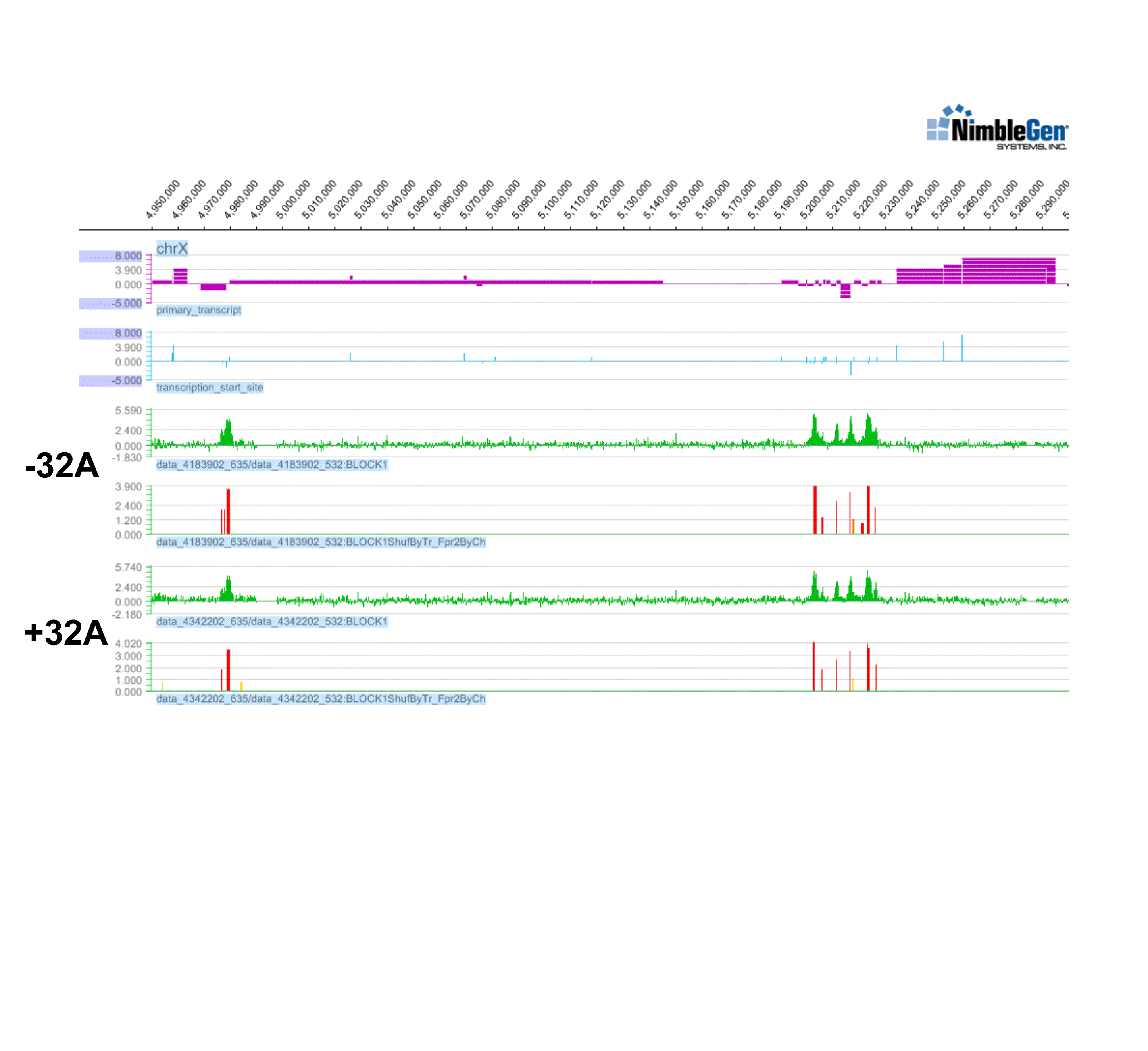

Supplement: Figure S5 — The panel shows an alignment of ChIP-on-chip analysis (graphs in green) using anti-BEAF antibodies that recognize the BEAF-32A splicing variant (‘+32A') or not (−32A). The red bars mark the position of significant peaks over the same region of the X chromosome (nucleotide positions 4,950,000 to 5,300,000) as shown in Figure 2. TSS (blue bars) and primary transcripts (purple lines) are shown on top. (1.4 MB TIF) [file pbio.0060327.sg005.tif]

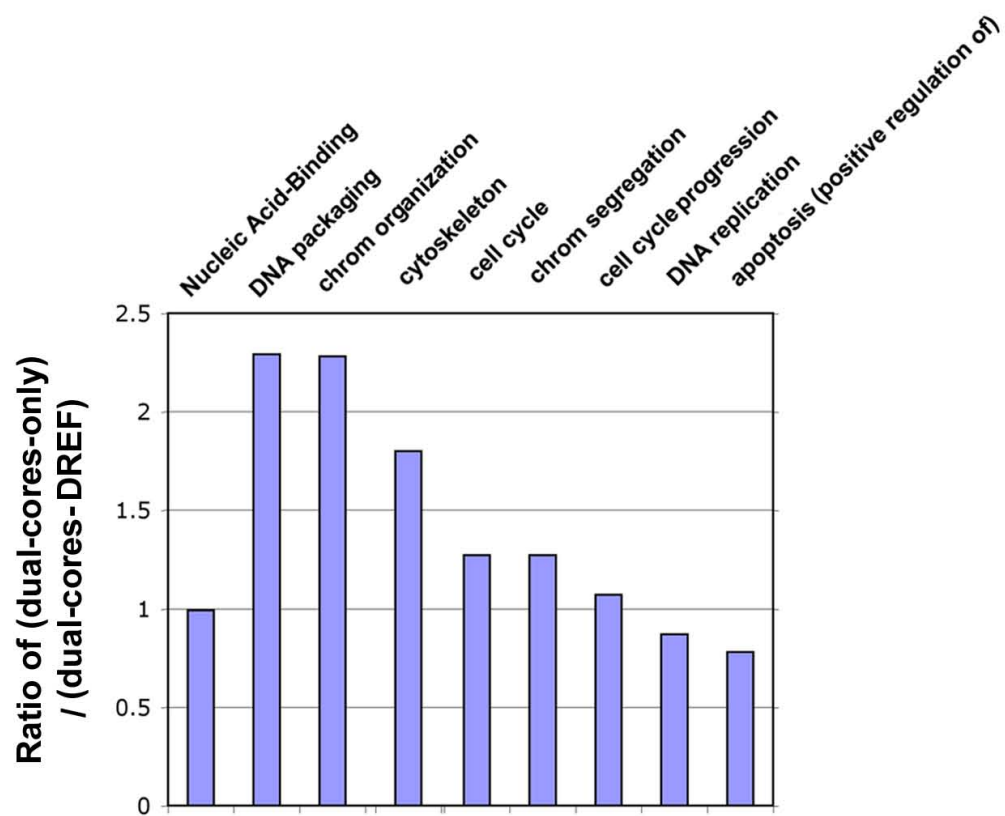

Supplement: Figure S6 — p-Values for gene annotations (GOs) of BEAF dual-cores-only (“dual-cores-only”) versus dual-cores containing additional TATCGATA consensus sites for DREF (“dual-cores-DREF”) [50]. The ratio of p-values is shown for each independent GO category and highlights a greater enrichment for BEAF dual-cores–only sites in chromosome organization (left) and for dual-cores–DREF sites in cell-cycle and apoptosis (right). DREF competes with BEAF for binding to a nested consensus sequence [34] present in dual-cores marked by a “_D” sign (see our Web site). These are significantly enriched in common GOs, including cell-cycle, in agreement with genetic interactions between beaf and dref [33,50] (see text for details). (124 KB PDF) [file pbio.0060327.sg006.pdf]

**A**

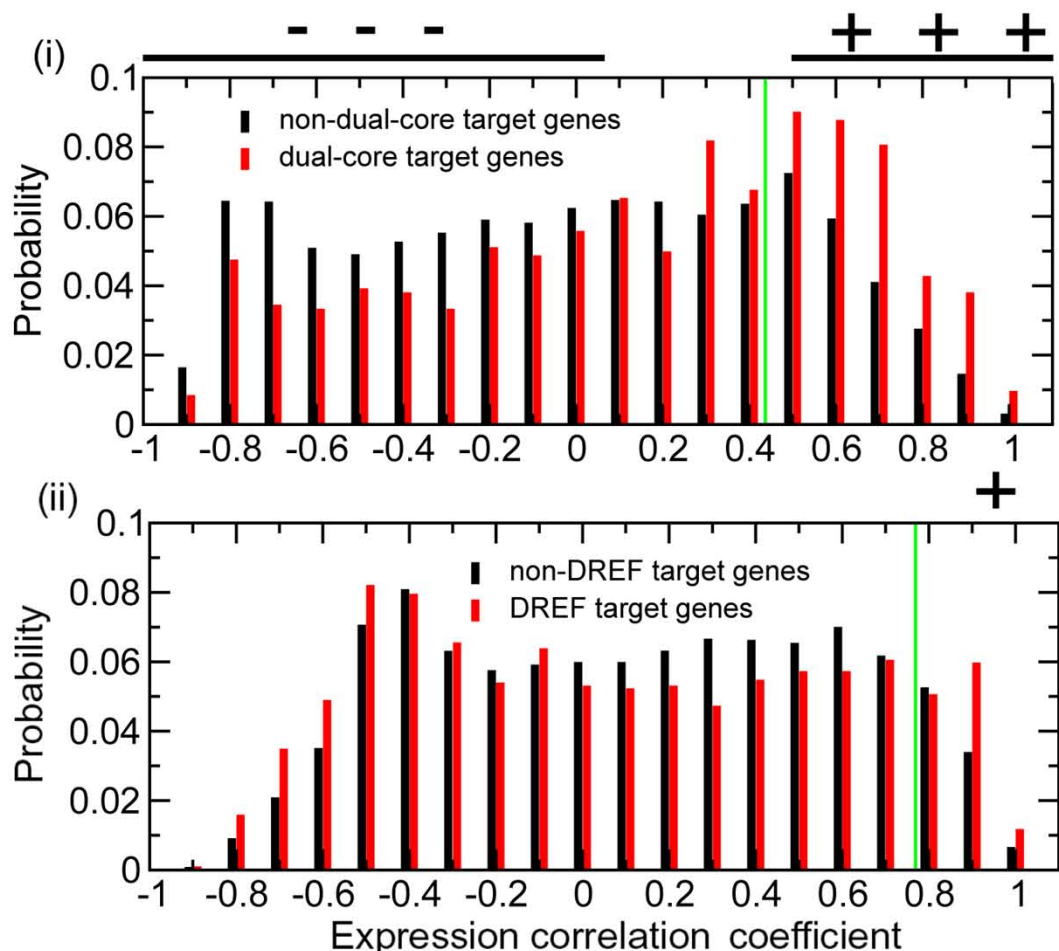

**B**

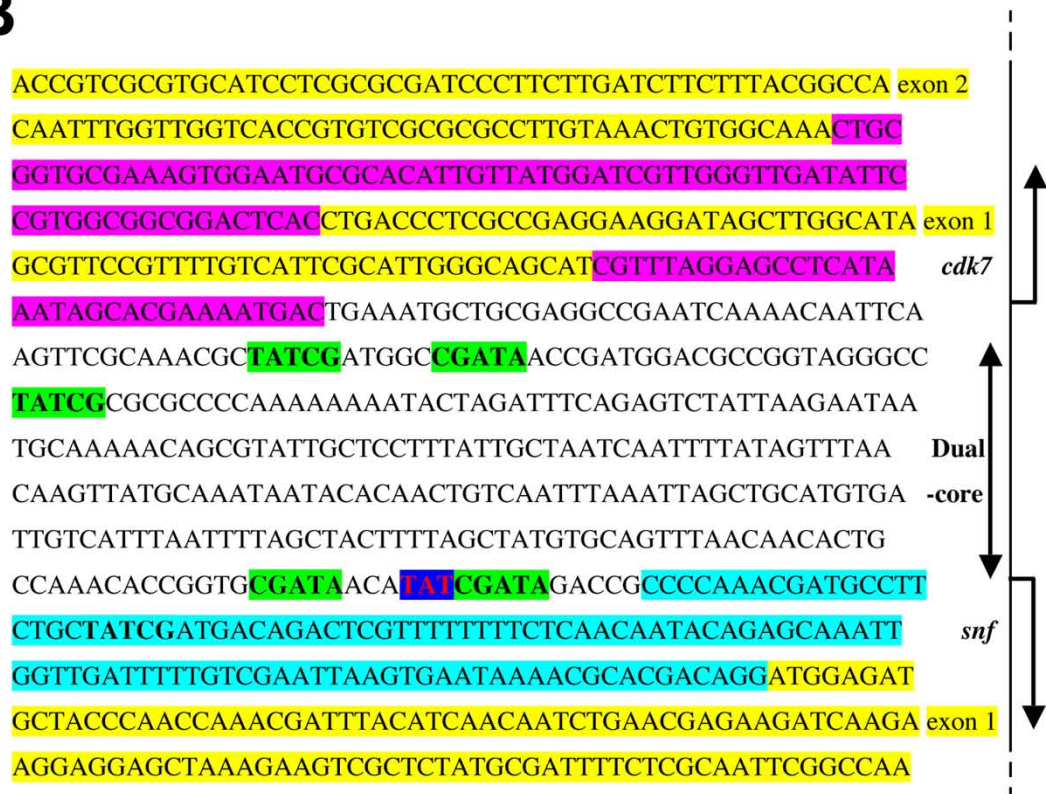

Supplement: Figure S7 — (A) BEAF dual-cores have a global positive impact on transcription. Distribution of correlation coefficients between the expression profile of genes with (red) or without (black) BEAF dual-cores (i) in their promoters (see Materials and Methods). “+” and “−” signs indicate statistical enrichment for co-regulated and anti-correlated gene expression profiles, respectively. As a positive control, the target genes for DREF [50] are enriched, as expected, for a minor subpopulation highly co-expressed with DREF (ii), but less significantly (p-value of 0.004 according to the Kolmogorov-Smirnov test) than BEAF, which has a more global positive effect on gene expression (p-value ∼ 3e-17 according to the Kolmogorov-Smirnov test). (B) Distribution of the BEAF (CGATA, green boxes) and DREF (TATCGATA, red) motifs in the Dual-core 38_D with respect to snf and cdk7 (TSS corresponds to the first colored bp). (309 KB PDF) [file pbio.0060327.sg007.pdf]

**A**

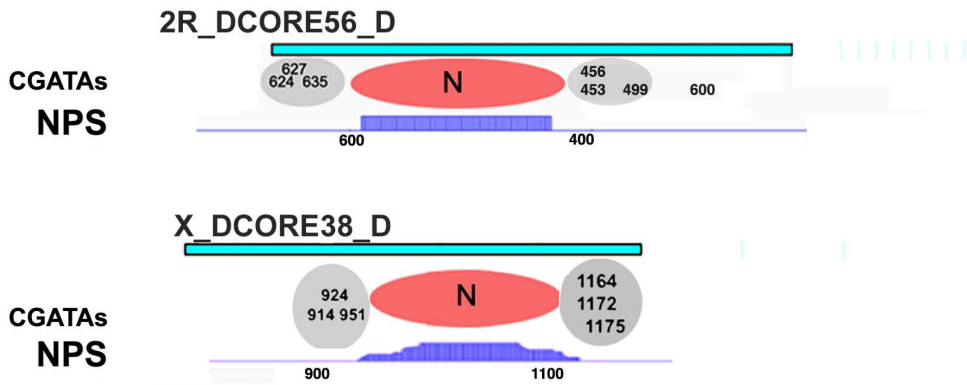

**B**

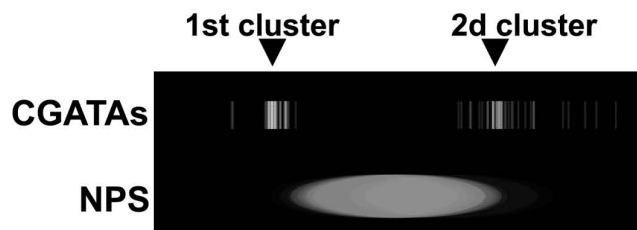

**C**

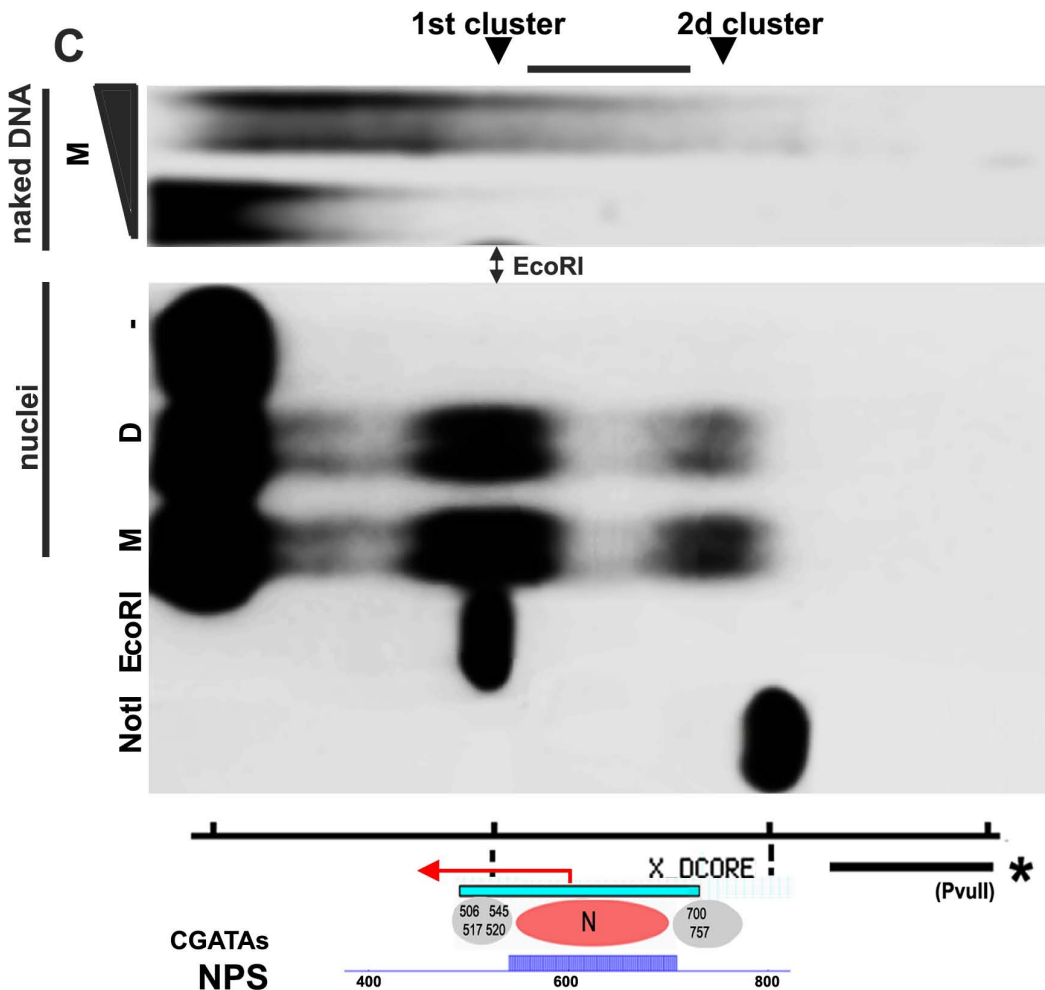

Supplement: Figure S8 — (A,B) Relative positioning of CGATA BEAF consensus binding motifs and the position of putative NPSs predicted by submitting dual-core sequences to available databases [42,43] in the cdk7 and mei-S332 promoter regions (A) as well as in >20 cell-cycle regulatory genes (B) (see our Web site for a list). Predicted NPSs are indicated by purple boxes below dual-cores (A) or as an overlay of predicted NPSs (B). The relative position of nuclease-resistant cores is indicated (N; according to experiments as shown in (C)). These predictions fit with the positions of AT-rich dual-core spacers (see Figure 1D). (C) Mapping of the accessibility of naked DNA control (top photograph) and of chromatin by nuclease digestion of nuclei (MNase,“M”; or DNAase I, “D”; see Materials and Methods). To map nuclease-resistant/sensitive regions with respect to CGATA clusters of dual-cores, purified genomic DNA was further digested with a second enzyme (PvuII +NotI or EcoRI) which cuts into the first CGATA cluster or 50 bp 3′ of the second CGATA cluster, respectively (see dotted lines below the autoradiogram). The dual-core spacer fits into a nuclease-resistant core region bracketed by hypersensitive sites. Note that these features are not found in the naked DNA control, where genomic DNA was first purified before MNase digestion. (239 KB PDF) [file pbio.0060327.sg008.pdf]
