# Supplementary material for: BEAF Regulates Cell-Cycle Genes through the Controlled Deposition of H3K9 Methylation Marks into Its Conserved Dual-Core Binding Sites
Source: PLoS Biol. 2008 Dec 23;6(12):e327. doi: 10.1371/journal.pbio.0060327 (PMC2605929; doi:10.1371/journal.pbio.0060327)
Supplement: Table S1 — GO terms for 1,720 BEAF dual-core target genes, which contain a dual-core within +/− 1,000 bp of their promoter.671 dual-core elements hit one promoter in the genome. The second column gives the number of annotated genes in that GO class, the third column gives the number of genes in dual-core/promoter sets in that GO class, the fourth column shows the expected number of genes in the predicted set given the observed class frequency. The corresponding p-value is given in the fifth column. GO terms have been binned into larger categories. Low-scoring GO classes underrepresented in the set of dual-core target genes are shown at the bottom. See our database for a complete listing and additional GO analysis. (25 KB DOC) [file pbio.0060327.st001.doc]

**Table S1**

**GO term # genes targets expect p-value biological process**

GO:0007049 483 66 33.95 0.00000041 cell cycle

GO:0016071 212 36 14.90 0.00000206 mRNA metabolism

GO:0007059 127 25 8.93 0.00000664 chromosome segregation

GO:0048468 355 49 24.96 0.00000994 cell development

GO:0019953 472 57 33.18 0.00007851 sexual reproduction

GO:0000278 278 38 19.54 0.00011631 mitotic cell cycle

GO:0000003 518 60 36.41 0.00015483 reproduction

GO:0051276 174 26 12.23 0.00036914 chromosome organization and biogenesis

GO:0006259 377 45 26.50 0.00054696 DNA metabolism

GO:0000902 350 42 24.60 0.00074508 cellular morphogenesis

GO:0007444 309 38 21.72 0.00084610 imaginal disc development

GO:0007281 118 19 8.30 0.00093036 germ cell development

GO:0006333 100 17 7.03 0.00095358 chromatin assembly or disassembly

GO:0008380 158 23 11.11 0.00109619 RNA splicing

GO:0048592 152 22 10.69 0.00149028 eye morphogenesis

GO:0007309 80 14 5.62 0.00198813 oocyte axis determination

GO:0001745 136 20 9.56 0.00199331 compound eye morphogenesis (sensu

Endopterygota)

GO:0000910 89 15 6.26 0.00200155 cytokinesis

GO:0006917 63 12 4.43 0.00205813 induction of apoptosis

GO:0006323 139 20 9.77 0.00254474 DNA packaging

GO:0006325 139 20 9.77 0.00254474 establishment and/or maintenance of chromatin

architecture

GO:0007455 149 21 10.47 0.00256186 eye-antennal disc morphogenesis

GO:0043065 65 12 4.57 0.00264394 positive regulation of apoptosis

GO:0035220 120 18 8.44 0.00265217 wing disc development

GO:0016331 85 14 5.98 0.00339277 morphogenesis of embryonic epithelium

GO:0035214 163 22 11.46 0.00341719 eye-antennal disc development

GO:0051242 163 22 11.46 0.00341719 positive regulation of cellular physiological

process

GO:0051301 145 20 10.19 0.00403684 cell division

GO:0019219 906 85 63.69 0.00471762 regulation of nucleic acid metabolism

GO:0012502 71 12 4.99 0.00525425 induction of programmed cell death

GO:0007560 246 29 17.29 0.00578607 imaginal disc morphogenesis

GO:0042981 110 16 7.73 0.00588072 regulation of apoptosis

GO:0007017 203 25 14.27 0.00593247 microtubule-based process

GO:0040007 91 14 6.40 0.00605257 growth

GO:0048513 657 64 46.19 0.00624321 organ development

GO:0050793 82 13 5.76 0.00630662 regulation of development

GO:0048024 55 10 3.87 0.00639249 regulation of nuclear mRNA splicing, via

spliceosome

GO:0009952 133 18 9.35 0.00739393 anterior/posterior pattern formation

GO:0048518 207 25 14.55 0.00746987 positive regulation of biological process

GO:0051252 57 10 4.01 0.00808694 regulation of RNA metabolism

GO:0051169 67 11 4.71 0.00898262 nuclear transport

GO:0007314 58 10 4.08 0.00905413 oocyte anterior/posterior axis determination

GO:0007391 77 12 5.41 0.00959528 dorsal closure

.

**Low-scoring GO classes**

**GO term # genes targets expect p-value biological process**

GO:0007600 266 10 18.70 0.99006747 sensory perception

GO:0042221 269 16 18.91 0.78189881 response to chemical stimulus

GO:0044262 278 17 19.54 0.75064793 cellular carbohydrate metabolism

GO:0007186 320 11 22.50 0.99756134 G-protein coupled receptor protein signaling

pathway

GO:0006118 336 19 23.62 0.85849720 electron transport

GO:0044255 337 21 23.69 0.74059949 cellular lipid metabolism

GO:0009308 379 24 26.64 0.72515723 amine metabolism

GO:0006812 390 24 27.42 0.77221097 cation transport

GO:0006807 394 25 27.70 0.72493983 nitrogen compound metabolism

GO:0006811 470 28 33.04 0.83667888 ion transport

GO:0005975 488 27 34.31 0.91680738 carbohydrate metabolism

GO:0006629 493 32 34.66 0.70099083 lipid metabolism

GO:0006952 516 32 36.27 0.78794248 defense response

GO:0050877 636 41 44.71 0.73604763 neurophysiological process

GO:0006508 764 52 53.71 0.61453336 proteolysis

GO:0050896 1192 83 83.80 0.55280469 response to stimulus

GO:0007165 1337 92 93.99 0.60132405 signal transduction
